# Glycosyl Phosphatidylinositol Anchor Biosynthesis Is Essential for Maintaining Epithelial Integrity during *Caenorhabditis elegans* Embryogenesis

**DOI:** 10.1371/journal.pgen.1005082

**Published:** 2015-03-25

**Authors:** Yemima Budirahardja, Thang Dinh Doan, Ronen Zaidel-Bar

**Affiliations:** 1 Mechanobiology Institute, National University of Singapore, Singapore; 2 Department of Biomedical Engineering, National University of Singapore, Singapore; University of California San Diego, UNITED STATES

## Abstract

Glycosylphosphatidylinositol (GPI) is a post-translational modification resulting in the attachment of modified proteins to the outer leaflet of the plasma membrane. Tissue culture experiments have shown GPI-anchored proteins (GPI-APs) to be targeted to the apical membrane of epithelial cells. However, the in vivo importance of this targeting has not been investigated since null mutations in GPI biosynthesis enzymes in mice result in very early embryonic lethality. Missense mutations in the human GPI biosynthesis enzyme *pigv* are associated with a multiple congenital malformation syndrome with a high frequency of Hirschsprung disease and renal anomalies. However, it is currently unknown how these phenotypes are linked to PIGV function. Here, we identify a temperature-sensitive hypomorphic allele of *PIGV* in *Caenorhabditis elegans*, *pigv-1(qm34)*, enabling us to study the role of GPI-APs in development. At the restrictive temperature we found a 75% reduction in GPI-APs at the surface of embryonic cells. Consequently, ~80% of *pigv-1(qm34)* embryos arrested development during the elongation phase of morphogenesis, exhibiting internal cysts and/or surface ruptures. Closer examination of the defects revealed them all to be the result of breaches in epithelial tissues: cysts formed in the intestine and excretory canal, and ruptures occurred through epidermal cells, suggesting weakening of the epithelial membrane or membrane-cortex connection. Knockdown of *piga-1*, another GPI biosynthesis enzymes resulted in similar phenotypes. Importantly, fortifying the link between the apical membrane and actin cortex by overexpression of the ezrin/radixin/moesin ortholog ERM-1, significantly rescued cyst formation and ruptures in the *pigv-1(qm34)* mutant. In conclusion, we discovered GPI-APs play a critical role in maintaining the integrity of the epithelial tissues, allowing them to withstand the pressure and stresses of morphogenesis. Our findings may help to explain some of the phenotypes observed in human syndromes associated with *pigv* mutations.

## Author Summary

Cell surface proteins, such as receptors, either integrate into the plasma membrane through a transmembrane domain or are tethered to it by an accessory glycosylated phospholipid (GPI) anchor that is attached to them after they are made. The GPI-anchor biosynthesis pathway is highly conserved from yeast to humans and null mutations in any of the key enzymes are lethal at early developmental stages. Point mutations in several genes encoding for GPI-anchor biosynthesis enzymes have been linked to human disease. Specifically, mutations in *PIGV* are associated with multiple congenital malformations, including renal and anorectal malformation and mental retardation. It is currently not known how the mutations in *PIGV* lead to these diseases. Here we describe a point mutation in the *PIGV* ortholog of the nematode *Caenorhabditis elegans*, *pigv-1*, which is found to cause a high degree of embryonic lethality. We documented a substantial reduction in the level of GPI-anchors in the mutant. Importantly, following its development using 4D microscopy and employing tissue-specific rescue, we identified loss of epithelial integrity as the primary cause of developmental arrest. Our results highlight the importance of GPI-anchored proteins for epithelial integrity in vivo and suggest a possible etiology for human diseases associated with *PIGV* mutations.

## Introduction

Proteins can attach to the plasma membrane by intrinsic transmembrane domains or by post-translational modifications with lipid moieties. One such lipid modification, tethering proteins to the outer leaflet of the plasma membrane (PM), is a glycosylphospatidylinositol (GPI) anchor, whose synthesis and attachment to proteins in the endoplasmic reticulum (ER) is a multi-step process involving >30 enzymes [1]. Proteins subjected to GPI anchor modification harbor two signal peptides, an N terminal signal peptide that targets them to the ER and a C terminal signal peptide that serves as a marker for GPI attachment [2]. The basic structure of a GPI anchor consists of a phosphoethanolamine linker, a glycan core and a phospholipid tail. The glycan core of GPI can be modified by other phosphoethanolamine or other sugar groups, giving rise to diverse GPI anchor structures [3].

From the ER, GPI-anchor proteins (GPI-APs) are transported to the Golgi, where the phospholipid tail of GPI anchor undergoes lipid remodeling to increase the efficiency of membrane binding. GPI-AP are then sorted and subsequently delivered to the outer leaflet of the PM through the trans-Golgi network [4]. GPI-APs are mostly localized to the apical membrane of polarized cells and are enriched in domains known as lipid rafts [5,6]. Extraction of lipid rafts using weak non-ionic detergent pulls down GPI-APs along with the rafts [7]. Apical polarization of GPI-APs has also been observed *in vivo* in epithelial cells of pancreas, intestine and urinary bladder in GFP-GPI-expressing mice. GPI-APs are also present in non-polarized tissues with equal distribution across the membrane [8].

GPI-APs have very diverse functions in various cells across species. They are required for viability and cell wall biosynthesis in yeast, act as defense against host immune system in trypanosome, mediate cell-cell interactions, signal transduction, and perform enzymatic activity in mammalian cells [1,3,9]. At the tissue level, GPI-APs were shown to be important for germline and oocyte development in the nematode *Caenorhabditis elegans (C*. *elegans)*. Mutation in *piga-1* (ortholog of mammalian *PIGA*), the catalytic subunit of phosphatidylinositol N-acetylglucosaminyltransferase complex, the first enzyme playing a role in GPI biosynthesis, decreases the number of germline mitotic cells and compromises oocyte formation and maturation [10]. At the organism level, GPI-APs were shown to be essential for mouse and human embryogenesis. A complete *PIGA* knockout mouse could never be obtained and this may be explained by the fact that mouse embryonic stem cells depleted of PIGA form embryoid bodies that are arrested at an early stage of differentiation [11,12].

While each individual GPI-AP has a unique function that depends on the protein itself, there is evidence to suggest that GPI anchors themselves, independent of the proteins they anchor, play a role in organizing the PM. Moreover, despite the fact that GPI-anchors are positioned in the outer leaflet of the PM, they have been shown to be affected by the organization of the actin cortex underlying the PM [13,14].

Mounting evidence supporting essential roles for GPI-APs during human embryogenesis comes from human genetic studies conducted in the past decade. Missense mutations in genes encoding enzymes catalyzing various steps of GPI anchor biosynthesis, such as PIGW that catalyzes attachment of acyl group to phosphatidylinositiol [15,16], PIGV that catalyzes transfer of second mannose to GPI intermediate [17,18], PIGT that attaches GPI to proteins [19] and PGAP2 that modifies the phospholipid tail of PI [20] result in congenital diseases known as hyperphosphatasia mental retardation syndrome (HPMRS), Hirschprung disease, morphological malformation and renal anomalies [21,22,23,24].

Studies on the roles of GPI-APs during embryogenesis have been hindered by the difficulties in obtaining viable mutants for GPI biosynthesis enzymes. In this study, we exploit a hypomorphic temperature-sensitive allele of *pigv-1* (human *PIGV* ortholog) in *C*. *elegans* to investigate the role of GPI-APs during embryogenesis. We found that GPI-APs are vital for the integrity of epithelial tissues during morphogenesis, suggesting an essential role for GPI-APs in stabilizing the apical membrane of epithelial tissues under stress.

## Results

### A temperature sensitive missense mutation in *pigv-1(qm34)* leads to defects in embryonic elongation

**Fig 1:**
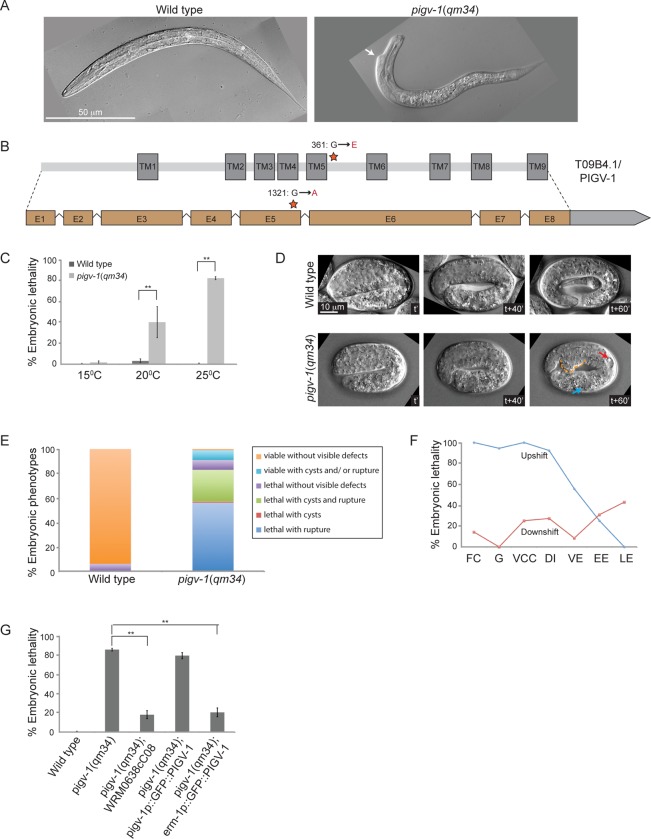
A temperature sensitive missense mutation in *pigv-1(qm34)* leads to defects in embryonic elongation. (A) Wild type L1 larva (left panel), and *pigv-1*(*qm34*) (right panel) exhibiting a morphological defect close to the head. (B) Gene and protein organization of PIGV-1. *qm34* is a *pigv-1* allele with a missense mutation at the amino acid 361 converting glycine to glutamic acid (indicated by a star). E denotes exon and TM denotes transmembrane. Light gray box after exon 8 indicates the 3’UTR. (C) *pigv-1*(*qm34*) embryos are heat-sensitive; Percent of wild type and *pigv-1*(*qm34*) embryos that fail to hatch at 15^°^C, 20^°^C, and 25^°^C. (D) Wild type embryo progressively elongates from 2 folds to almost 4 folds in an hour (upper panel), whereas *pigv-1*(*qm34*) embryo arrested during elongation at 3 fold stage (lower panel) with a rupture (stippled orange line) and cysts formed in excretory canal (red arrow) and intestine (blue arrow). In all images, anterior is to the left, time stamp is in minutes. (E) Classification of the terminal phenotypes of wild type and *pigv-1*(*qm34*) embryos based on 4D Nomarski movies, as shown in D. (F) In the upshift experiments, *pigv-1*(*qm34*) embryos at different embryonic stages dissected from gravid adults grown at 15^°^C were upshifted to 25^°^C and maintained at the restrictive temperature throughout embryogenesis and scored as lethal when they did not hatch at the end of embryogenesis. For downshift experiments, similar procedure was done except that worms were kept at 25^°^C for 24 hours before downshifted to 15^°^C. FC denotes founder cells generation, G denotes gastrulation, VCC denotes ventral cleft closure, DI denotes dorsal intercalation, VE denotes ventral enclosure, EE denotes early elongation and LE denotes late elongation. (G) WRM0638cC08 fosmid containing *pigv-1* and GFP::PIGV-1 expression driven by *erm-1* promoter significantly rescues *pigv-1*(*qm34*) embryonic lethality. Data in C and G are presented as average ± s.e.m. For each experiment, more than 100 embryos were counted. Experiments were done in duplicate and repeated five times (*P <0.05, **P<0.01, two-tailed test). See S1, S2, S3 and S4 Tables for numerical values and statistical analyses of data presented in panel C, E, F and G, respectively.

In the course of our whole genome sequencing of maternal-effect morphologically abnormal (*mal*) mutants isolated by Hekimi et al. [25] in an ethyl methanesulfonate (EMS) mutagenesis screen, we discovered that *mal-3*(*qm34*) (Fig. 1A) has a missense mutation at amino acid 361 of previously unassigned gene T09B4.1, converting glycine to glutamate (Fig. 1B). BLAST analysis of the T09B4.1 protein sequence suggested that it is an ortholog of human GPI mannosyltransferase 2, which is known as *PIGV* (S1A–B Fig.). The mutation was verified by conventional sequencing, and from this point onwards we refer to *mal-3*(*qm34*) as *pigv-1*(*qm34*).

Sometimes, EMS mutagenesis results in hypomorphic alleles that are temperature-sensitive. We investigated this possibility by growing *pigv-1*(*qm34*) worms at 15, 20 and 25^°^C and measuring their viability at each temperature. We found that pigv-1(*qm34*) is a heat-sensitive allele, with more than 80% embryonic lethality at 25°C (Fig. 1C, S1 Table). We followed embryogenesis by time-lapse differential interference contrast (DIC) microscopy and observed phenotypes resulting from *pigv-1* inactivation at 25^°^C. At this temperature *C*. *elegans* embryogenesis takes 10.5 hours from the first division till hatching (S2 Fig.). The first 3 hours of embryogenesis are characterized by formation of the founder cells, rapid cell division and gastrulation. At around 3 hours the epidermis is born on the dorsal side of the embryo and the next 3.5 hours are dominated by epidermal morphogenesis, a three step process made up of intercalation, enclosure, and elongation [26]. Loss of *pigv-1* resulted in defects appearing during elongation with cysts forming inside the embryo and/or cells leaking out from the embryo body, resulting in elongation arrest and embryonic lethality (Fig. 1D). Quantification of 176 *pigv-1(qm34)* embryos showed that over 80% displayed ruptures and/or cyst formation and arrested in elongation (Fig. 1E). Few escapers hatched and became L1 larva with body shape defects (Fig. 1A).

Utilizing the heat sensitivity of *pigv-1*(*qm34*), we determined the developmental period when *pigv-1* activity is required through reciprocal temperature shift experiments. We found that *pigv-1* activity is essential from the one cell embryo stage until elongation. Once elongation was underway inactivation of PIGV-1 had less effect on embryogenesis (Fig. 1F, S3 Table).

We confirmed that these phenotypes are caused by the mutation in *pigv-1* by rescue experiments. Transformation of *pigv-1*(*qm34*) worms with a fosmid that contains a wild-type allele of the *pigv-1* gene significantly rescued embryonic lethality (*P*<0.01) (Fig. 1G, S4 Table). Expression of a *gfp*-tagged *pigv-1* under the control of 2.4 kb upstream of the *pigv-1* start codon failed to rescue embryonic lethality in *pigv-1*(*qm34*) mutant worms, most likely due to low expression. On the other hand, expression of *pigv-1* under the control of the *erm-1* promoter, which resulted in 3 fold stronger expression, successfully rescued embryonic lethality of *pigv-1*(*qm34*) (Fig. 1G, S4 Table), confirming that the mutated gene causing lethality in the *pigv-1*(*qm34*) strain is *pigv-1*.

### GPI-APs are present throughout embryogenesis and are enriched in apical membranes of epithelial cells after differentiation

**Fig 2:**
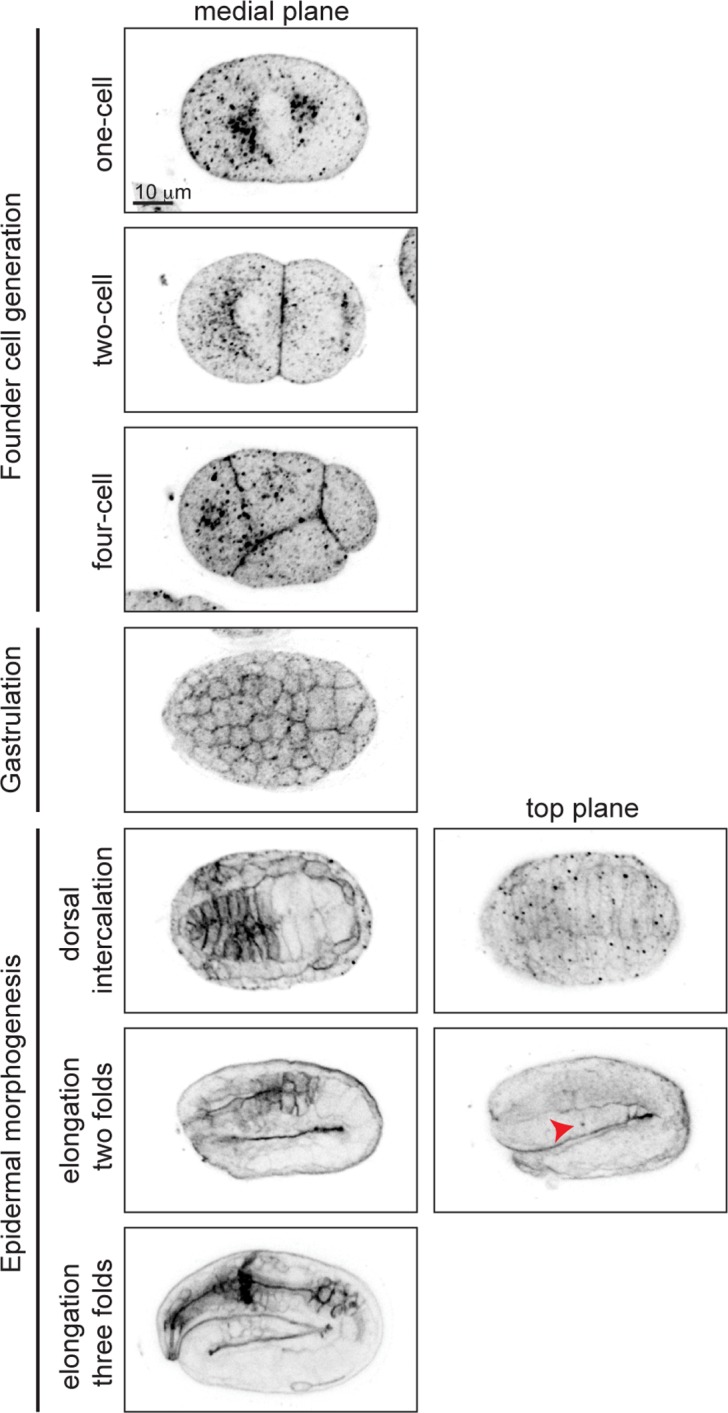
GPI-APs are present at the membrane throughout embryogenesis. Proaerolysin coupled to Alexa 488 (FLAER) was used to visualize GPI-APs in wild type embryos. GPI-APs are highly enriched at pharyngeal membranes, initially non-polarized, but then becomes polarized at the apical membrane during elongation. Red arrowhead denotes excretory canal.

To visualize GPI-AP distribution during embryogenesis, we used Alexa-488 labeled proaerolysin (FLAER), a bacterial toxin that binds specifically to GPI-AP [27], to label embryos at different stages of development (Fig. 2). In the one-cell embryo, GPI-APs accumulated at perinuclear areas and were enriched in the anterior cytoplasm (Fig. 2, first row). As soon as new membrane was delivered to the cell surface, during cell division, GPI-APs accumulated on the plasma membrane (Fig. 2, second row). During gastrulation we observed GPI-APs accumulated at the membrane of all cells (Fig. 2, fourth row). While being uniformly localized on membrane of all cells in the early embryo, non-uniform GPI-AP distribution was observed upon tissue differentiation. For example, during dorsal intercalation, GPI-APs were highly enriched on pharyngeal cell membranes in a non-polarized manner, whereas later on, during elongation, they became apically enriched (Fig. 2, fifth to seventh rows).

### PIGV-1 is expressed in all embryonic epithelial tissues where it is required for GPI-anchor biosynthesis

**Fig 3:**
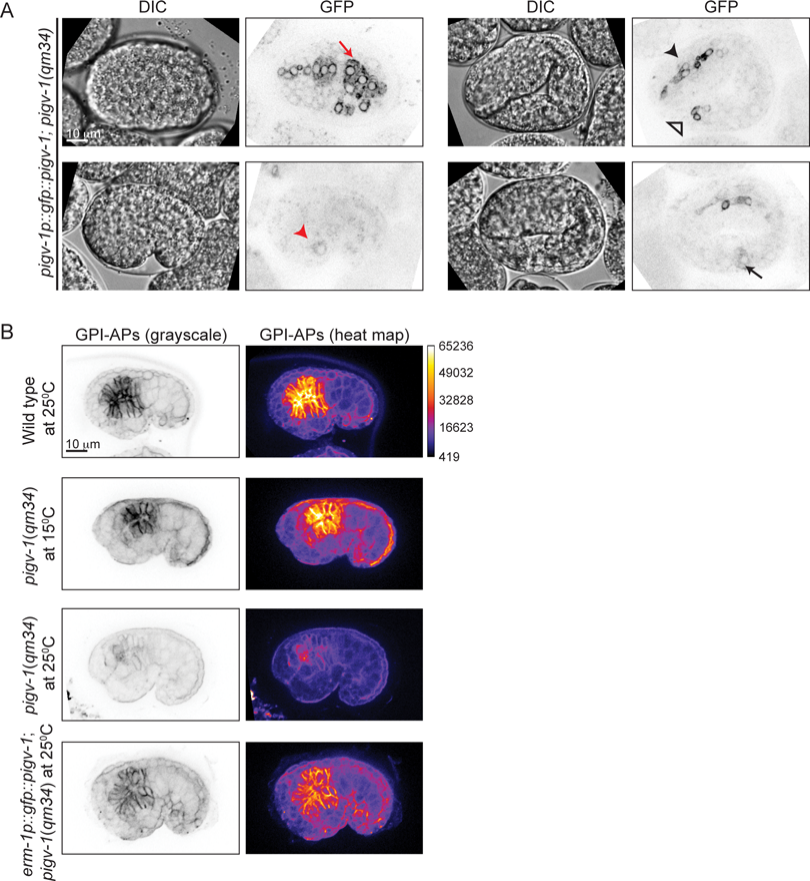
PIGV-1 is expressed in all embryonic epithelial tissues where it is essential for GPI-anchor biosynthesis. (A) PIGV-1 is expressed in epithelial tissues during embryogenesis: epidermis (top left panel); excretory cell (bottom left panel); pharynx, and rectum (top right panel) and intestine (bottom right panel). In this and subsequent images, red arrow indicates epidermis, black arrow indicates intestine, black arrowhead indicates pharynx and red arrowhead indicates excretory canal and open arrowhead indicates rectum. (B) FLAER staining highlights GPI-APs localization at the membrane of embryonic tissues in wild type and mutant embryos at comma stage. Differences in FLAER staining intensity are demonstrated by heat maps of corresponding images (right panels). Similar fluorescence intensity is observed in wild type and *pigv-1*(*qm34*) embryos grown at the permissive temperature, but highly reduced in *pigv-1*(*qm34*) embryos grown at the restrictive temperature. Expressing PIGV-1 in *pigv-1*(*qm34*) restores GPI-AP biosynthesis and their localization at restrictive temperature.

To gain insight into the spatial and temporal activity of the PIGV-1 enzyme during embryogenesis, we visualized an N-terminally GFP-tagged PIGV-1 driven by its endogenous promoter in an extrachromosomal array. We could not detect GFP::PIGV-1 in the early embryo, possibly due to silencing of the transgenes in the germline. Later in development the expression level was low. Nevertheless, we observed GFP::PIGV-1 to be prominent in the epidermis, pharynx, intestine, rectum and excretory cell (Fig. 3A), all tissues with epithelial character. At the subcellular level, PIGV-1 localized to intracellular structures that appear to be ER [28,29].

Using FLAER staining as readout for GPI-anchor biosynthesis, we compared the intensity of FLAER staining in *pigv-1(qm34)* at the permissive (15°C) and restrictive (25°C) temperatures. At 15°C, *pigv-1*(*qm34*) embryos exhibited FLAER levels similar to wild type, whereas at 25°C, abrogation of PIGV-1 activity led to a 4-fold reduction in the FLAER signal (Fig. 3B, second and third rows). FLAER staining was restored to wild-type levels in *pigv-1*(*qm34*) embryos at 25°C when *pigv-1* was expressed in all epithelial tissues by the *erm-1* promoter (Fig. 3B, fourth row). Taken together these data show that GPI-APs are enriched in epithelial tissues and the abundance of GPI-APs at the cell membrane is dependent on the activity of PIGV-1.

### Loss of function of *piga-1* exhibits phenotypes similar to *pigv-1(qm34)* mutant embryos

**Fig 4:**
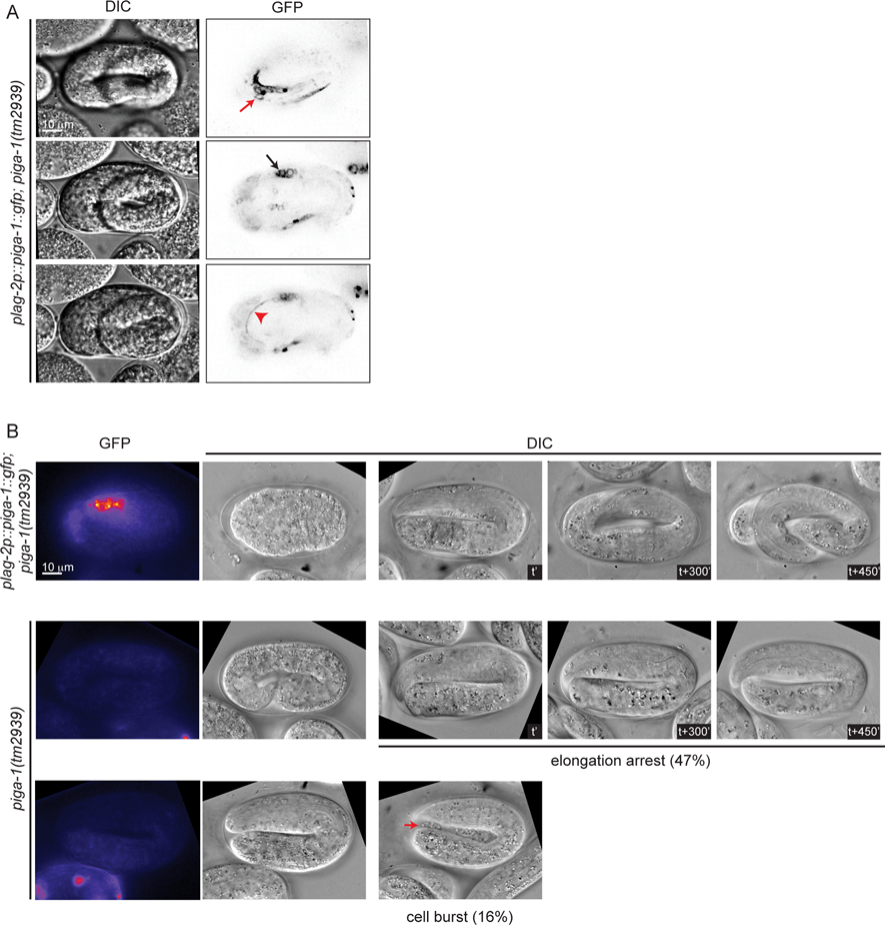
Loss of function of *piga-1* shows similar phenotypes to *pigv-1(qm34)* mutant embryos. (A) *plag-2* promoter drives *piga-1* expression ubiquitously in various tissues: epidermis (top panel), intestine (middle panel) and excretory canal (bottom panel). (B) Loss of PIGA-1 expression results in elongations defects. Embryos were dissected from gravid adults expressing *plag-2*::*piga-1*::*gfp* and examined for their expression and phenotypes during embryogenesis. Those retaining PIGA-1::GFP expression elongate and hatch (top panel), whereas ~50% of embryos losing PIGA-1::GFP expression arrest during elongation with or without rupture (middle and lower panels).

In mammalian cells, more than 30 enzymes are known to regulate the GPI anchor biosynthesis pathway. Many of these enzymes have orthologs in *C*. *elegans* (S1B Fig.). A previous study has shown that RNAi-mediated knockdown of most *C*. *elegans* GPI anchor biosynthesis enzymes does not lead to any phenotype and two of them, namely *pigk and pigo* resulted in sterility [10]. We scanned a range of feeding RNAi conditions for *pigk* and *pigo*, with the rationale that partial loss of function might bypass their requirement for germline development, and expose a possible role in embryogenesis. However, we observed either sterility or no phenotype when each of the two enzymes was depleted. Thus, we turned our attention towards *piga-1*(*tm2939*) mutant worms characterized in the previous study [10]. Progeny of homozygous *piga-1*(*tm2939*) worms are embryonic lethal, and they display a deformed eggshell due to increased osmotic sensitivity during germline development. To uncouple the functions of PIGA-1 during germline development and embryogenesis, we used *piga-1*(*tm2939*) worms rescued by *piga-1* expression under the control of *lag-2*, a distal tip cell promoter. First, we examined whether *lag-2* drives *piga-1* expression during embryogenesis and found *piga-1* expressed ubiquitously during embryogenesis (Fig. 4A). Since the *plag-2*::*piga-1*::*gfp* construct is expressed as an extrachromosomal array, some embryos lose *piga-1* expression during embryogenesis. We identified which embryos lost the extrachromosomal array and followed their embryonic phenotypes. While all the embryos retaining *piga-1* expression during embryogenesis hatched, ~50% of the embryos devoid of *piga-1* expression were arrested during elongation. In one-third of arrested embryos, internal cells leaked out from the embryo body (Fig. 4B), a phenotype reminiscent of *pigv-1*(*qm34*) embryos, suggesting that weakening of epithelial tissue integrity is not a specific phenotype of *pigv-1* loss of function, but rather a general consequence of disruption of the GPI biosynthesis pathway.

### Loss of GPI-APs leads to breaches in epithelial tissue in the epidermis, embryonic intestine and excretory canal

The elongation phase of *C*. *elegans* embryogenesis is characterized by the formation of circumferential actin bundles (CFB) in the dorsal and ventral epidermal cells and actomyosin contraction in the lateral epidermal cells [30]. Contractility of the muscle tissues is known to be required for elongation beyond the 2-fold length [31]. We tested whether the elongation arrest occurring upon *pigv-1* inactivation is caused by defective CFB and/or muscle organization. We examined CFB in *pigv-1*(*qm34*) embryos using an F-actin reporter (VAB-10 actin binding domain tagged with GFP) and found that CFB structure is indistinguishable from that of wild type embryo (S3A Fig.). Myotactin antibodies were utilized to examine muscle organization and we observed no difference between muscle organization in wild type and in *pigv-1*(*qm34*) embryos (S3B Fig.). Moreover, some *pigv-1*(*qm34*) embryos elongated beyond two-fold stage. These results suggest that elongation arrest in *pigv-1*(*qm34*) embryos is not caused by defects in CFB or muscle structure.

**Fig 5:**
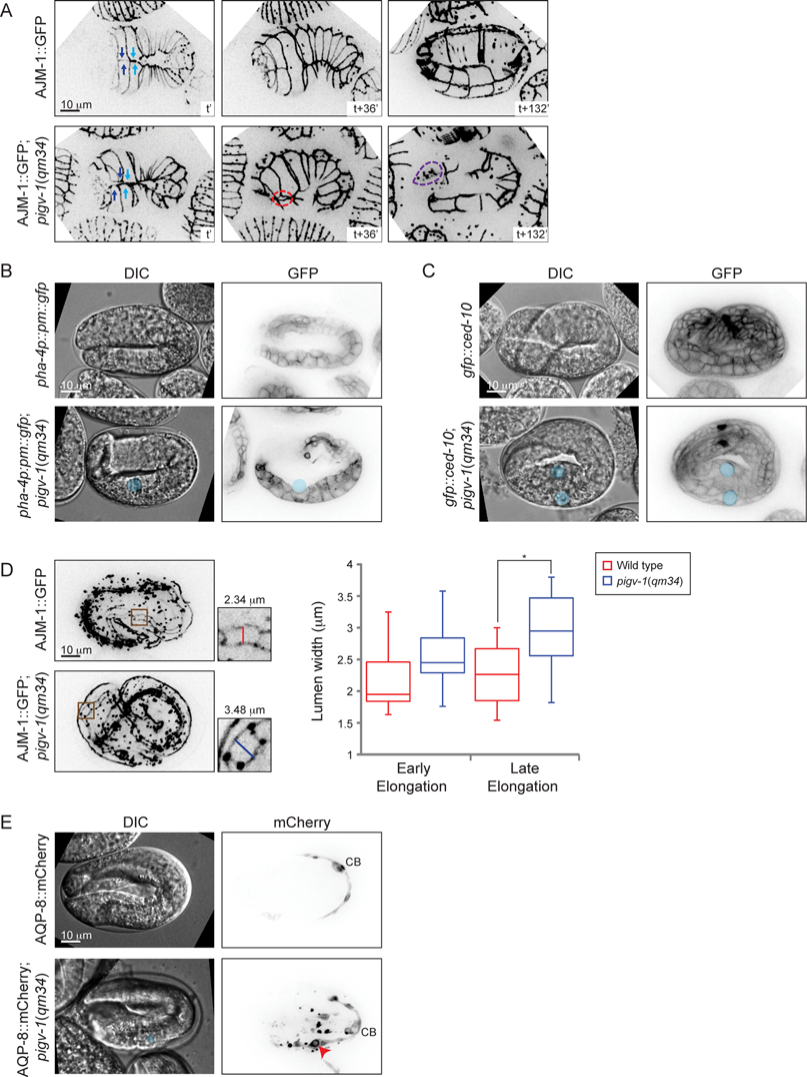
Loss of GPI-APs leads to breaches in epithelial tissue in the embryonic epidermis, intestine and excretory canal. (A) Epidermal cell sealing at the embryo ventral midline is defective in *pigv-1*(*qm34*) mutant. In wild type embryo, the two pairs of leader cells coming from left and right sides of the embryo perfectly align at the embryo midline (top left panel), leading to embryo enclosure (top middle panel) and followed by successful elongation (top right panel). In *pigv-1*(*qm34*) mutant, two pairs of ventral epidermal leader cells misalign at embryo midline (bottom left panel). As a result, a gap is formed between the leader cells (bottom middle panel) and subsequently cells are bursting out from the hole (bottom right panel). See the corresponding movies S1 Movie and S2 Movie. Cell junction in all panels is marked by AJM-1::GFP. A maximum intensity projection of embryo epidermis is shown in all panels. Light and dark blue arrows indicate two pairs of leader epidermal cells, stippled red circle indicates gap between leader cells and stippled magenta circle indicates location of rupture. (B) Cysts form at intestine basal side in *pigv-1*(*qm34*) embryo. Blue circle indicate cyst location in both DIC and GFP panels. (C) Cysts are located in between intestine and basal lamina in *pigv-1*(*qm34*) embryo. Blue circles indicate cyst location in both DIC and GFP channels. (D) Intestinal lumen is enlarged in *pigv-1*(*qm34*) embryo. Wild type and *pigv-1*(*qm34*) embryos expressing AJM-1::GFP and undergoing elongation were fixed. A maximum intensity projection of embryo intestine was made and the widest section of intestine both in wild type and in *pigv-1*(*qm34*) embryos were measured (inset). The graph shows lumen width in wild type and *pigv-1*(*qm34*) embryos at early elongation (2 and 2.5 folds), as well as late elongation (3 and 3.5 folds). The line inside the box indicates median, whereas the whiskers indicate minimum and maximum values. Lumen width in *pigv-1*(*qm34*) embryo is significantly wider than that of wild type at late elongation (n > 7 for each data set, *P <0.05, two-tailed test) (E) Cyst forms in excretory canal of *pigv-1*(*qm34*) embryo. Excretory canal is marked by AQP-8::mCherry. Blue circle indicates cyst location in DIC channel, red arrowhead indicates cyst location in mCherry channel and CB stands for cell body. A maximum intensity projection of the excretory canal is shown in mCherry channels.

We then set out to characterize the embryonic phenotypes resulting from *pigv-1* inactivation in more detail. We employed several cell junction and membrane markers expressed in specific tissues to pinpoint the location of the defects. Using AJM-1::GFP and HMP-1::GFP as a marker for epidermal apical junctions, we observed gaps between epidermal cells through which internal cells leaked out, most often from the embryo anterior (Fig. 5A and S1–S4 Movies). In some embryos, the gap is created by misalignment of leading ventral epidermal cells coming from opposite ends to enclose the embryo at the ventral midline (Fig. 5A). Using a plasma membrane marker specifically expressed in the pharynx and intestine, we identified cysts to be located at the basal side of the intestine, and using CED-10::GFP, which highlights plasma membrane of all cells, we observed cysts to be located between the intestine and its surrounding basal lamina (Fig. 5B-C, S4 Fig.). Using AJM-1::GFP to highlight the apical junctions of intestinal cells we observed widening of the lumen in *pigv-1*(*qm34*) embryos (Fig. 5D). We measured intestinal lumen width of wild type and *pigv-1*(*qm34*) embryos in early (2–2.5 fold) and later (3–3.5 fold) stages of elongation and found that the lumen width of *pigv-1*(*qm34*) embryos was significantly wider (P<0.05) than that of wild type embryos at late elongation (Fig. 5D). Furthermore, we noticed that the intestine in *pigv-1*(*qm34*) embryos was often twisted (S6C Fig.).

Using mCherry-tagged AQP-8, a water channel specifically localized to the excretory canal, expressed at a low level which maintains a normal translumenal flux, we found the excretory canal to be another location where cysts formed in *pigv-1*(*qm34*) embryos (Fig. 5E). In contrast with the intestinal cysts that formed in extracellular space the excretory canal cysts formed within the cell. Few embryos that survived embryogenesis hatched with excretory canal cysts (S5 Fig.). The excretory canals in larvae with excretory canal cysts were usually very short. Not only the length, but the branching of the excretory canal is also affected in *pigv-1*(*qm34*) embryo. In wild-type worms the excretory canal extends four tubules shaped like an H: a pair towards anterior and another pair towards posterior from the cell body. However, the excretory canal in *pigv-1*(*qm34*) embryo often has one or two more tubules extending from the cell body or branching from the original tubules (S7 Fig.).

GPI-APs are known to be targeted to apical membranes in polarized epithelial cells [5,6]. We therefore examined whether the apicobasal polarity of epithelial cells is affected in *pigv-1*(*qm34*) embryos. We observed that the apical markers PAR-6 and PKC-3 were correctly localized on the apical intestinal and excretory cell membranes in *pigv-1*(*qm34*) embryos (S6A Fig.), and AJM-1 was localized at the apical side of epidermal and intestinal cell-cell junctions (S6B–C Fig.). Conversely, the basolateral marker LET-413 was localized to the basolateral membranes in epidermal and intestinal cells in *pigv-1*(*qm34*) embryos, indistinguishable from wild type (S6B Fig.). Similarly, the intermediate filament IFB-2 was correctly localized beneath the apical membrane in intestinal tissue in *pigv-1*(*qm34*) embryos (S6C Fig). Altogether, these results rule out a polarity defect as the underlying cause for the *pigv-1*(*qm34*) mutant phenotypes.

### GPI-AP biosynthesis in all epithelial tissues is required to rescue embryonic lethality of *pigv-1(qm34)*

**Fig 6:**
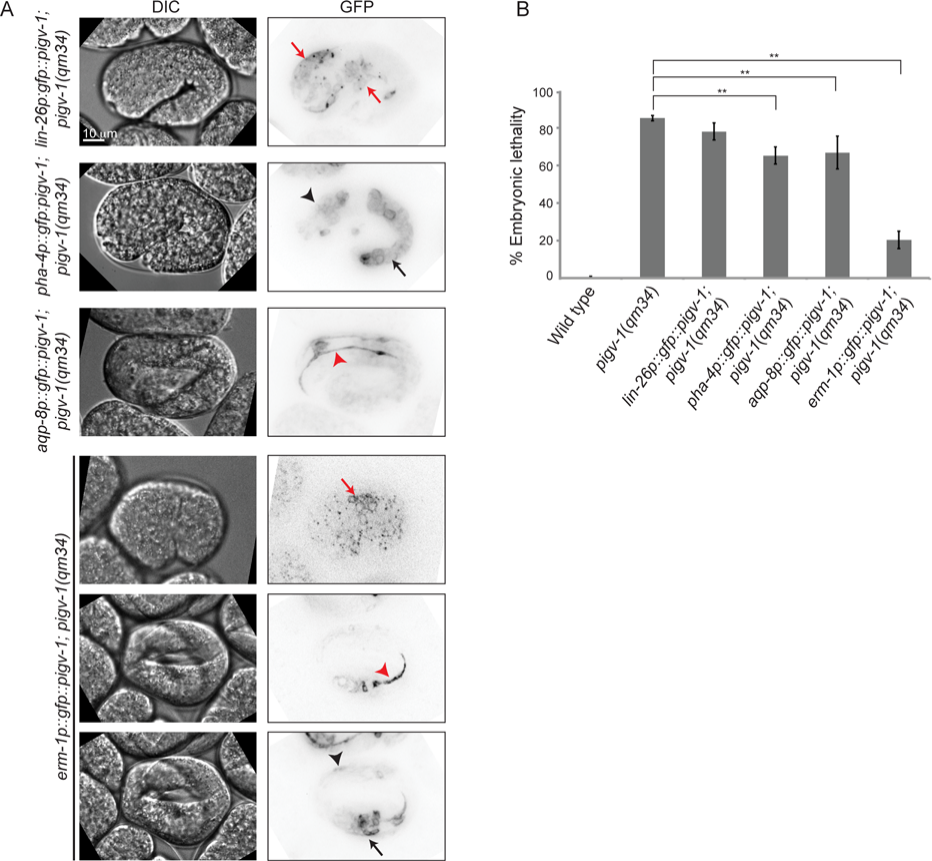
GPI-AP biosynthesis in epithelial tissues is sufficient to rescue embryonic lethality of *pigv-1(qm34)*. (A) Several epithelial tissue specific promoters were utilized to express *pigv-1* in *pigv-1*(*qm34*) embryos: *lin-26* promoter for epidermal expression (first panel); *pha-4* promoter for pharyngeal and intestinal expression (second panel); *aqp-8* promoter for excretory canal expression (third panel); *erm-1* promoter for all epithelial tissue expression (fourth to sixth panel). A sum projection of embryonic tissues is shown in all panels. (B) PIGV-1 expression in each epithelial tissue rescues *pigv-1*(*qm34*) embryonic lethality at various degrees, whereas PIGV-1 expression in all epithelial tissues driven by erm-1 promoter rescues *pigv-1*(*qm34*) embryonic lethality the highest. Data are presented as average ± s.e.m. For each experiment, more than 100 embryos were counted. Experiments were done in duplicate and repeated five times (**P<0.01, two-tailed test). See S4 Table for numerical values and statistical analyses.

We observed *pigv-1* loss of function to affect the integrity of three epithelial tissues: epidermis, intestine and excretory canal. However, it was not immediately evident which defective tissue was responsible for the embryonic lethality. To address this question we restored *pigv-1* expression specifically in each epithelial tissue or in all epithelial tissues of *pigv-1(qm34)* worms and determined their embryonic viability. We employed the *lin-26* promoter to drive expression in the epidermis, the *pha-4* promoter to drive expression in the pharynx and intestine, the *aqp-8* promoter to drive expression in the excretory canal, and the *erm-1* promoter to drive expression in all epithelial tissues (Fig. 6A). All promoters drove *pigv-1* expression at comparable levels.

While restoring *pigv-1* expression in the pharynx-intestine or in the excretory canal partially reduced *pigv-1*(*qm34*) embryonic lethality, restoring *pigv-1* expression in the epidermis did not significantly reduce *pigv-1*(*qm34*) embryonic lethality (Fig. 6B, S4 Table). The most significant rescue of embryonic lethality achieved by expression of *pigv-1* in a single tissue was a 17% reduction in lethality. In contrast, expressing *pigv-1* in all epithelial tissues using the *erm-1* promoter sharply decreased *pigv-1*(*qm34*) embryonic lethality down by 62%, comparable to the sum of the embryonic rescue of each epithelial tissue (Fig. 6B, S4 Table). Thus, it appears that *pigv-1* function is required in all epithelial tissues for embryonic viability.

### Overexpression of ERM-1::GFP rescues embryonic lethality of *pigv-1(qm34)*

**Fig 7:**
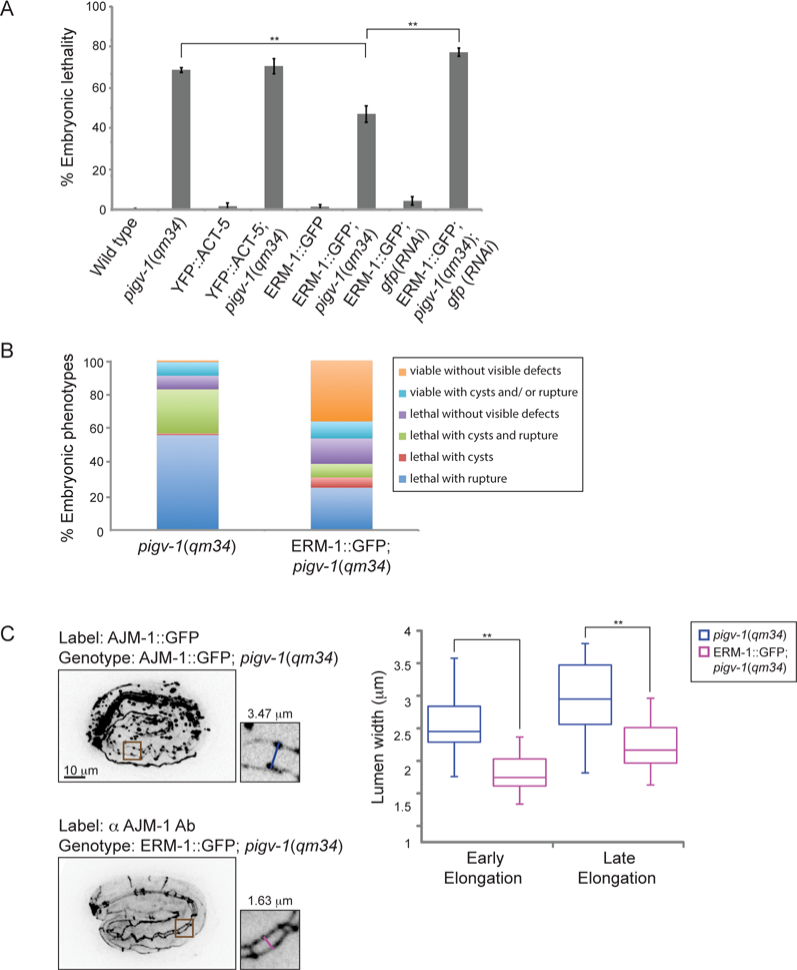
Overexpression of ERM-1::GFP rescues embryonic lethality and intestinal lumen width of *pigv-1(qm34)*. (A) Embryonic lethality of *pigv-1*(*qm34*) is suppressed by ERM-1::GFP, but not by YFP::ACT-5 overexpression. Data are presented as average ± s.e.m. For each experiment, more than 100 embryos were counted. Experiments were done in duplicate and repeated five times (**P<0.01, two-tailed test). (B) Overexpression of ERM-1::GFP rescues *pigv-1*(*qm34*) embryonic lethality phenotypes. (C) Overexpression of ERM-1::GFP rescues enlarged intestinal lumen width in *pigv-1*(*qm34*) embryos (n>10, **P<0.01, two-tailed test). See S2 and S5 Tables for numerical values and statistical analyses.

The cytoskeletal cortex underlying the plasma membrane provides it with structural support and protects the membrane from mechanical stress. Depletion of spectrin in erythrocytes changes membrane rigidity and subsequently leads to cell fragmentation [32]. Thus, we hypothesized that strengthening the actin cortex in *pigv-1*(*qm34*) embryos might positively affect membrane integrity. First, we examined whether providing more actin has any impact on epithelial membrane integrity. We examined *pigv-1*(*qm34*) embryos overexpressing YFP::ACT-5 in the epidermis and intestine and found that embryonic lethality in these worms is indistinguishable from that of *pigv-1*(*qm34*) (Fig. 7A, S5 Table). We then explored whether strengthening the link between the actin cortex and the cell membrane might influence membrane integrity. We chose worms overexpressing ERM-1::GFP at a level which does not cause any phenotypic defect since a previous study showed that at high levels of expression ERM-1 leads to formation of excretory canal cysts [33]. We crossed the ERM-1::GFP-overexpressing worms with *pigv-1*(*qm34*) and found that embryonic lethality was significantly reduced in the *pigv-1(qm34)*;ERM-1::GFP strain. Depletion of overexpressed ERM-1 by *gfp*(*RNAi*) in this stain reverted embryonic lethality back to *pigv-1*(*qm34*) level, confirming ERM-1 overexpression is responsible for rescuing *pigv-1*-associated embryonic lethality (Fig. 7A, S5 Table). Careful examination of embryogenesis in *pigv-1*(*qm34*) embryos overexpressing ERM-1::GFP revealed strong suppression of *pigv-1* phenotypes, and a significant portion of embryos (36%) hatched without any visible defects (Fig. 7B, S2 Table). Considering ERM-1 localization at intestine and excretory canal apical membranes, we reasoned that ERM-1 overexpression could rescue apical-associated phenotypes in these tissues. Measuring the width of intestinal lumen we found that it was reduced to the wild type dimension (Fig. 7C).

**Fig 8:**
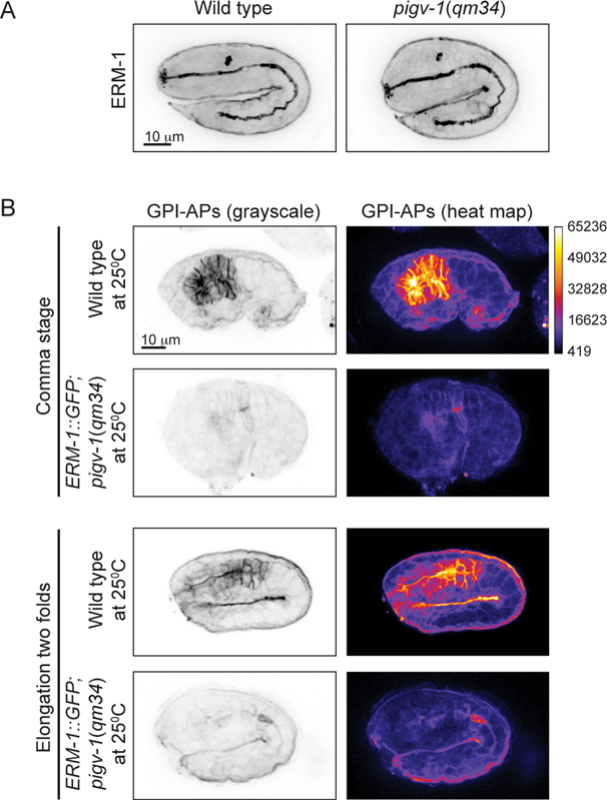
ERM-1 overexpression does not rescue GPI-AP biosynthesis in *pigv-1*(*qm34*). (A) Endogenous ERM-1 distribution and level are not affected in *pigv-1*(*qm34*) embryo. A maximum intensity projection of embryo intestine is shown for wild type and *pigv-1*(*qm34*) embryos. (B) ERM-1::GFP overexpression does not restore GPI-AP levels at the membrane. The level of GPI-APs in *pigv-1*(*qm34*) embryos expressing ERM-1::GFP at restrictive temperature (second and fourth panels) is > 10 folds lower than that of wild type embryo (first and third panels).

To gain insight into the mechanism of *pigv-1*-phenotype rescue by ERM-1 overexpression, we examined whether endogenous ERM-1 distribution and level were altered in *pigv-1*(*qm34*) embryos. Immunolabeling with ERM-1 antibodies showed no difference in ERM-1 distribution or level between wild type and *pigv-1*(*qm34*) embryos (Fig. 8A). We then asked whether ERM-1 might rescue *pigv-1* mutant by enhancing the residual *pigv-1* activity and restoring the level of GPI-APs. Using FLAER as the probe for GPI-APs, we found that GPI-AP level in *pigv-1(qm34)* embryos overexpressing ERM-1 is similar to *pigv-1(qm34)* embryos alone, suggesting that ERM-1 overexpression does not rescue *pigv-1* embryonic lethality by restoring GPI-APs (Fig. 8B).

## Discussion

GPI anchor is an important post-translational protein modification whose functions and mechanisms have been widely studied using unicellular organisms and mammalian cells in culture [1,6,9]. However, the role of GPI biosynthesis in animals remains poorly understood. In humans, somatic mutations in *PIGA* gene loci lead to paroxysmal nocturnal hemaglobinuria, a disease characterized by increased susceptibility of erythrocytes to lysis by the complement immune system [34]. No heritable mutation in *piga* gene in human has been identified, suggesting that *PIGA* function is required during embryogenesis. Indeed, deletion of *PIGA* gene in mice, which completely abrogates GPI biosynthesis, resulted in early embryogenesis defects [11,35]. However, this condition precludes the study of GPI function throughout embryogenesis.

Also in *C*. *elegans*, a null mutation in *piga-1* results in germline defects and early embryonic lethality. In this study, we circumvented the early requirements for GPI-APs by using a temperature sensitive hypomorphic allele of *pigv-1*. The amount of GPI-APs remaining upon *pigv-1* inactivation was sufficient for normal germline development, thus enabling us to uncover their requirement during embryogenesis. We showed that GPI-APs are present and function throughout embryogenesis. Interestingly, the phenotypes of *pigv-1* inactivation, i.e., weakened epithelial tissues, are manifested only late in embryogenesis during the elongation stage of epidermal morphogenesis. This may be due to increased mechanical forces generated by actomyosin in muscle and epidermis tissues at that stage.

Although present at the membrane of all cells in the *C*. *elegans* embryo, *pigv-1* loss of function exhibits no defect in early development events, such as gastrulation or tissue differentiation. This is in contrast to mammalian embryogenesis, in which complete PIGA depletion results in defects in tissue differentiation [35]. One possible explanation for this difference is that the residual GPI-APs in *pigv-1* animals are sufficient for normal differentiation. Another reason could be differences in the proteins regulating differentiation. Tissue differentiation in mammals is regulated by BMP/ TGF-β signaling whose activation requires GPI-anchored co-receptors, Dragon and Cripto-1 [35,36]. Although present in *C*. *elegans*, BMP/TGF-β signaling is not required during embryogenesis, but operates during postembryonic development, regulating body size [37,38].

Inactivation of *pigv-1* in *C*. *elegans* embryos resulted mainly in epithelial defects. Failures in epidermal enclosure and intestinal cyst formation are consistent with weaker cell-cell adhesion. In the epidermis, improper cell-cell adhesion creates gaps between ventral epidermal cells from which internal tissues leak out during elongation. Compromised cell-cell junctions in the intestine, which has higher osmotic pressure than the surrounding tissues, would allow passage of low molecular weight substances, such as water molecules, from the intestinal lumen to the intestine basal side. The presence of a basal lamina separating the intestine from the pseudocoelom results in accumulation of these substances in the form of cysts.

One possible explanation of these results is that the reduction in the amount of one or more specific cell adhesion proteins that are GPI-anchored causes the observed defects. However, amongst the GPI-APs that have been experimentally identified in *C*. *elegans* none are known to mediate cell-cell adhesion [7,10]. While we do not rule out the involvement of yet unknown GPI-anchored adhesion proteins, we propose another mechanism to explain the observed epithelial phenotypes that does not depend on a specific protein, but rather on the GPI anchors themselves. Goswami et. al. have shown that cortical actin affects the organization of GPI-AP in the membrane [13]. We propose that GPI-APs are enriched in apical membranes of polarized epithelial cells where they play a role in organizing the membrane into domains that interact with the actin cortex within the cell and through these interactions stabilize the apical membrane. According to this model, a decrease in GPI-APs will lead to fewer membrane-cortex connections and thus to a weaker apical membrane. In support of this idea, we observed a widening of the intestinal lumen in *pigv-1* mutant embryos, as would be expected if the apical membrane of the intestine is weakened and thereby cannot resist as well the osmotic pressure from inside the lumen. Further support for this model comes from the finding that overexpression of ERM-1 rescues lumen width and overall embryonic lethality of *pigv-1* mutant embryos. ERM-1, the sole *C*. *elegans* ortholog of ezrin, radixin and moesin, is a linker protein that has an actin-binding domain and attaches to the PM through its FERM domain, serving to connect the PM with the actin cortex [39,40]. ERM-1 overexpression did not increase the levels of GPI-APs in *pigv-1* embryos and hence the reduction in lethality associated with it is most likely due to its membrane-cortex cross-linking function. From this we deduce that loss of GPI-APs leads to a weakening of apical membranes in epithelial cells, irrespective of the proteins attached to the GPI-anchor.

Another epithelial tissue affected by *pigv-1* loss of function is the excretory canal. Down regulation of GPI-APs in the excretory canal leads to cysts formation and this cystic excretory canal is usually short, consistent with apical membrane weakening upon the loss of GPI-APs. Unlike the intestine, which is a multicellular tubule, the excretory canal is a unicellular tubule that extends actively during embryo elongation. It has been demonstrated that a balance between membrane-actin cortex recruitment and translumenal flux is essential for the excretory canal extension [33]. Weakened apical membrane upon down regulation of GPI-APs in *pigv-1* mutant embryo may prevent further recruitment of membrane components and actin undercoat to extend the canal, creating an imbalance between the two forces. Consequently, the dominant force, the translumenal flux is transmitted to enlarge the canal diameter, resulting in cysts formation. Another phenotype we observed in the excretory canal of pigv-1 mutants is ectopic branching. To our knowledge such a phenotype has not been associated with loss of function of any gene so far, opening a new avenue to study the regulation of tubular branching.

Besides epithelial tissues, loss of GPI-APs in *C*. *elegans* may also affects neuronal and/or muscle tissues, suggested by the lethargic phenotype of *pigv-1*(*qm34*) worms, although the GPI-APs responsible for this phenotype remains to be identified. The short excretory canal that has been observed occasionally in *pigv-1*(*qm34*) could also result from the loss of GPI-APs from neuronal membrane. The neuronal cell adhesion molecules (NCAM) that are essential for axon outgrowth and pathfinding have been demonstrated to regulate excretory canal extension [41,42,43]. In the absence of NCAM, the excretory canal does not grow to full extent. Supporting this view, the *in vitro* and the *in silico* experiments found several NCAMs (*rig-3*, *rig-6*, *rig-7* and *wrk-1*) to be potentially GPI-modified [7,44].

Not all epithelial tissues displayed abnormal phenotypes in *pigv-1* mutants. The pharynx and rectum are two epithelial tissues that do not seem to be affected by down regulation of GPI-APs. High enrichment of GPI-APs at pharyngeal membranes compared to other tissues could provide an explanation for the absence of weakened membrane phenotypes. However, this reason does not hold for the rectum, as GPI-APs at rectal membranes are not more abundant than other tissues that display weakened membrane. Since both pharynx and rectum are covered by a cuticle, the most likely explanation for the absence of visible phenotypes is that the cuticle protects both tissues from potential damage resulting from weakened membranes.

Restoring PIGV-1 expression individually in the epidermis, intestine and excretory canal in *pigv-1*(*qm34*) embryos revealed that the defects in these epithelial tissues do not contribute equally to the embryonic lethality. The intestine and the excretory canal defects have higher contribution to embryonic lethality as compared to the epidermal defects. This is somewhat unexpected because 56% of *pigv-1*(*qm34*) embryos die due to tissues leakage from the embryo interior, indicating that a gap between epidermal cells is present from where the tissues pass through. However, uncontained high pressure built in the intestinal and excretory canal due to cell adhesion defects and membrane weakening may be sufficient to open epidermal junction and push the internal tissues out of embryo interior.

Mutations in the human ortholog of *C*. *elegans pigv-1*, *PIGV*, have been associated in genetic studies with hyperphosphatasia-mental retardation syndrome (a.k.a Mabry syndrome). This autosomal recessive syndrome has a wide spectrum of phenotypes including intellectual disabilities, facial anomalies, hyperphosphatasia, vesicoureteral and renal anomalies, and anorectal anomalies [21]. With the exception of hyperphosphatasia, which is known to be the result of loss of GPI-anchored complement inhibitors in blood cells [34], the proteins and cellular functions that are affected in humans with *PIGV* mutations are unknown. Although our findings in *C*. *elegans* cannot possibly fully explain the cellular physiology of the human disease, it does point to a basic mechanism, i.e., weakening of apical membranes in epithelial cells, that may be playing a role in some of the manifestations of the disease. Furthermore, if it will be discovered that epithelial membrane integrity is affected in human patients then our work also suggests a promising avenue for therapy, i.e., strengthening of the membrane-cortex connection, based on our ERM-1 overexpression results.

## Materials and Methods

### Strains and alleles

Strains were grown and maintained at 20^°^C under standard conditions [45]. Wild type strain N2 was used as a control. The *pigv-1*(*qm34*) was retrieved from an EMS screening conducted by Hekimi et al. [25]. For analysis using GFP fusions, F2 progeny exhibiting *pigv-1* phenotypes and carrying the markers were selected from crosses between *pigv-1*(*qm34*) and the following strains: SU93 *jcIs1[ajm-1*::*gfp*, *unc-29(+)*, *rol-6p*::*rol-6(su1006)]* [46], SU265 *jcIs17[hmp-1p*::*hmp-1*::*gfp*, *dlg-1p*::*dlg-1*::*dsRed*, *rol-6p*::*rol-6(su1006)]* [47], SU467 *pIs7[pha-4p*::*pm*::*gfp*, *rol-6p*::*rol-6(su1006)]* [48], FT17 *xnIs3[par-6p*::*par-6*::*gfp*, *unc-119(+)]*; *unc-119(ed3) III*, MOT63 *temIs59[pIC26*::*pkc-3]*; *unc-119(ed3) III*, WS4918 *opIS310[ced-1p*::*yfp*::*act-5*::*let-858 3'UTR*, *unc-119(+)]* [49], VJ402 *fgEx1[erm-1p*::*erm-1*::*gfp*, *rol-6p*::*rol-6(su1006)]* [33], ML1735 *mcIs50[lin-26p*::*vab-10(actin-binding domain)*::*gfp*, *myo-2p*::*GFP]* [50], *plag-2p*::*piga-1*::*egfp*-expressing strain was generated by Murata et al [10].

### Plasmid construction

All plasmids generated in this study were constructed in a modified pPD95.75 backbone. For tissue-specific rescue of *pigv-1* loss of function, GFP position was changed to be at the N terminal instead of at the C terminal of the multiple cloning sites (MCS), whereas for AQP-8-expressing plasmid, GFP at C terminal was replaced with mCherry. To construct *pigv-1p*::gfp::pigv-1 plasmid, *pigv-1* promoter (2.4 kb sequence upstream of *pigv-1* start codon) and coding sequence were amplified and inserted into *Sbf*I and *Age*I sites upstream of *gfp* in original pPD95.75 vector. Circular PCR was performed to amplify the whole plasmid, but the *gfp* region using a pair of primers harboring *Xho*I sites at their 5’ ends. PCR product was then ligated to produce a circular plasmid containing *pigv-1* promoter and coding sequence, but without *gfp*. Second circular PCR was conducted to insert two new restriction sites, i.e.: *Not*I and *Bgl*II between *pigv-1* promoter and coding sequence. PCR product was then subjected to digestion using *Not*I and *Bgl*II. *gfp* coding sequence was amplified from original pPD95.75 and subcloned into pJET (Thermo Scientific). The recombinant plasmid was digested using *Not*I and *Bgl*II and *gfp* sequence-containing product was ligated to *pigv-1*-containing pPD95.75, resulting in a plasmid expressing *gfp*::*pigv-1* driven by *pigv-1* promoter. Four different promoters were used to rescue *pigv-1(qm34)* in different tissues: 4.1 kb sequence of *lin-26* promoter to drive *pigv-1* expression in epidermis, 7.1 kb of *pha-4* promoter for expression in pharynx and intestine, 2.2 kb of *aqp-8* promoter for expression in excretory canal and 3 kb of *erm-1* promoter for expression in all epithelial tissues. They are inserted into modified pPD95.75 at *Sbf*I/*Not*I sites replacing *pigv-1* promoter. Transgenic animals generated by injecting the constructs into the gonad of hermaphrodite animals resulted in the following strains: RZB40 (*pigv-1*(*qm34*); *msnEx40*[*lin-26p*::*gfp*::*pigv-1*; *rol-6*(*su1006*)]), RZB41 (*pigv-1*(*qm34*); *msnEx41*[*pha-4p*::*gfp*::*pigv-1*; *rol-6*(*su1006*)]), RZB129 (*pigv-1*(*qm34*); *msnEx129*[*aqp-8p*::*gfp*::*pigv-1*; *rol-6*(*su1006*)]) and RZB128 (*pigv-1*(*qm34*); *msnEx128*[*erm-1p*::*gfp*::*pigv-1*; *rol-6*(*su1006*)]).

To construct *aqp-8*::*mCherry*-expressing plasmid, mCherry coding sequence was amplified from pAA64 and ligated to circularly amplified pPD95.75 devoid of gfp sequence using Gibson assembly (NEB). Subsequently, 2.2 kb *aqp-8* promoter together with *aqp-8* genomic sequence were inserted at SbfI/BamHI sites in modified pPD95.75. Injection of this construct resulted in strain RZB221 (*pigv-1*(*qm34*); *msnEx221*[*aqp-8p*::*aqp-8*::*mCherry*; *rol-6*(*su1006*)]).

### Microinjection

Microinjection was performed as described by Mello and Fire [51]. Injection mix includes 100 μg/μl salmon sperm DNA digested with *Pvu*II, 20 μg/μl *rol-6*(*su1006*) digested with *Sbf*I and 5–10 μg/μl each construct digested with *Sbf*I.

### Whole genome sequencing and mutation validation

Genomic DNA was extracted from *pigv-1*(*qm34*) mutant worms using standard method and subjected to whole genome sequencing using Illumina platform and annotated using MAQGene [52]. The whole genome sequencing and its annotation were performed by Hobert lab (Columbia University). Candidate genes altered in *pigv-1*(*qm34*) were narrowed down using genetic mapping results done by Hekimi et al. [25]. Point mutation in *pigv-1* gene was confirmed by amplification of *pigv-1* gene in *pigv-1*(*qm34*) mutant worms, subcloning into pJET vector (Thermo Scientific) and followed by conventional sequencing (First Base). Further validation of *pigv-1* missense mutation as the phenotype-causing gene in *pigv-1*(*qm34*) worms was done by injection of 100 μg/μl fosmid WRM063BcC08, which contains *pigv-*1 gene, together with the co-transformation marker *rol-6*(*su1006*) into the gonad of *pigv-1*(*qm34*) hermaphrodites. F2 rollers were upshifted to 25^°^C and examined for embryonic lethality.

### Quantification of embryonic lethality

Ten to fifteen gravid hermaphrodites were placed on the plate and incubated for several hours to lay more than 100 eggs. Hermaphrodites were then removed and the number of eggs laid was counted. Twenty-four hours later, the number of larvae hatched was determined. Each experiment was done in duplicate and repeated five times. Beside experiments determining temperature sensitivity that are conducted at three different temperatures (15^°^C, 20^°^C and 25^°^C), the remaining experiments were conducted solely at 25^°^C to get the highest extent of *pigv-1* inactivation. In this case, L4 larvae were upshifted from 20^°^C to 25^°^C for 20 to 24 hours prior to the test.

### Temperature shift experiments

For upshift experiment, embryos were dissected from gravid *pigv-1*(*qm34*) worms grown at 15^°^C and incubated at 25^°^C for the duration of embryogenesis. Each embryo was staged and scored for hatching. For downshift experiment, similar procedure was performed, except that *pigv-1*(*qm34*) worms were kept at 25^°^C for 24 hours before downshifted to 15^°^C. Embryos that do not hatch at the end of embryogenesis were considered as lethal.

### 4D microscopy and Pigv-1 phenotype classification

Larvae or embryos collected from gravid hermaphrodite, mounted onto 3% agarose padded-glass slide, closed with coverslip and sealed with wax. Normaski images shown in Fig. 1A, B and S2B were captured using a Nikon Ti Eclipse widefield microscope equipped with DIC 1.40NA oil condenser and a charged-coupled device camera Cool Snap HQ_2_ (Photometrics). All other imaging were done using spinning disk confocal system composed of a Nikon Ti Eclipse microscope with a CSU-X1 spinning disk confocal head (Yokogawa), DPSS-Laser (Roper Scientific) at 491 and 568 nm excitation wavelength and an Evolve Rapid-Cal electron multiplying charged-coupled device camera (Photometrics). For both microscopes, Metamorph software (Molecular Devices) was used to control acquisition. Projected images were created using Fiji.

After 24 hour DIC recording, wild type, *pigv-1*(*qm34*) and *pigv-1*(*qm34*) embryos expressing ERM-1::GFP were scored as viable or lethal and each category is further classified into four subcategories; i.e.: without visible defects, with cysts and rupture, with cyst only and with rupture only.

### GFP knockdown by RNAi

IPTG plate used for *gfp*(*RNAi*) feeding was prepared as described [53]. Wild type and *pigv-1*(*qm34*) L1 larvae expressing ERM-1::GFP were fed using bacterial-feeding strain of *gfp* for 3 days at 15^°^C till they become L4 and then upshifted to 25^°^C for overnight. The absence of GFP signal was verified by using fluorescent stereomicroscope and only those devoid of the signal were subjected for embryonic lethality test.

### FLAER staining and immunolabeling

Fixation and indirect immunofluorescence were performed essentially as described [54]. The following primary mouse antibodies were used: ERM-1 (DSHB; 1/20), AJM-1 (MH27, DSHB; 1/10), myotactin (MH46, DSHB; 1/5) and LET-413 (DSHB; 1/2) and IFB-2 (MH33, DSHB; 1/5). Donkey anti-mouse coupled to Alexa 647 (1/500) (Life technologies) was used as secondary antibodies and proaerolysin coupled to Alexa 488 (FLAER, Protox Biotech) was used to detect GPI-APs. Images were taken on a Nikon Ti Eclipse spinning disk microscope with 100x objective and processed further using Fiji. To measure lumen width in wild type and *pigv-1*(*qm34*) mutant embryos, N2 and *pigv-1*(*qm34*) embryos expressing AJM-1::GFP were fixed, maximum intensity projection of embryonic intestine in GFP channel was constructed and the widest section of intestinal lumen was determined. The same procedure was done to measure lumen width in p*igv-1*(*qm34*) embryos expressing ERM-1::GFP, except that AJM-1 antibodies were used instead of AJM-1::GFP expression.

### Statistical analysis

Statistical analyses were done using Microsoft Excel. Two-tailed Student’s *t*-test was applied to compare the values.

## Supporting Information

S1 Fig T09B4.1 is an ortholog of human PIGV. (A) An alignment of human PIGV and *C*. *elegans* PIGV-1 shows a high degree of similarity between them. Amino acid residues are colored based on ClustalX color scheme (http://www.jalview.org/help/html/colourSchemes/clustal.html) and displayed using Jalview. (B) The GPI-anchor biosynthesis pathway in mammalian cells. The orthologous enzymes present in *C*. *elegans* are shown in black and PIGV-1 whose mutant allele is assessed in this study is highlighted in red.
(TIF)

S2 Fig The timeline of *C*. *elegans* embryogenesis at 25°C. *C*. *elegans* embryogenesis is initiated by multiple rounds of cell divisions that form the founder cells. Three germ layers are then formed subsequently during gastrulation. The end of gastrulation is marked by closure of a ventral cleft. All those events occur within the first 2.5 hours of embryogenesis. In the next 4 hours, the embryo undergoes epidermal morphogenesis, in which epidermal cells born at the dorsal side of embryo intercalate and enclose the embryo at the ventral side. The embryo then elongates to form worm-like shape and hatches.
(TIF)

S3 Fig Loss of GPI-anchor proteins does not affect epidermal circumferential actin bundles or muscles. (A) Organization of circumferential actin bundles (CFBs) in *pigv-1*(*qm34*) embryo is similar to wild type embryo. VAB-10 actin binding domain tagged with GFP is used to visualize CFBs. (B) Muscle organization and structure are indistinguishable between wild type and *pigv-1*(*qm34*) embryos. Myotactin antibodies (MH46) are utilized to display muscles. A maximum intensity projection of CFBs and muscles are shown in panel A and B, respectively.
(TIF)

S4 Fig Intestinal cysts are visibly distinct from the germ cells. The germ cells (GC) are distinguishable from other cell types in the embryo based on their round shape and paired localization (left panels). The GCs are visible in *pigv-1*(*qm34*) embryo in a separate focal plane from that of the intestinal cysts (middle and right panels). The cysts in *pigv-1*(*qm34*) embryo grow in size over time. GCs are colored in orange and cysts are colored in blue.
(TIF)

S5 Fig *pigv-1*(*qm34*) L1 larva has a short cystic excretory canal. While the excretory canal in wild type L1 larva is elongated (top panel), the excretory canal in *pigv-1*(*qm34*) L1 larva is cystic and short (bottom panel). In both DIC and mCherry channels, cyst is colored in blue.
(TIF)

S6 Fig Loss of GPI-anchor proteins does not affect epithelial polarity. (A) GFP::PAR-6 and GFP::PKC-3 are localized to pharynx and intestine apical membranes both in wild type and *pigv-1*(*qm34*) embryos. Stippled circle denotes excretory cell. (B) AJM-1::GFP and LET-413 are correctly localized to apical and basolateral membrane of epidermis, pharynx and intestine, respectively, both in wild type and *pigv-1*(*qm34*) embryos. (C) IFB-2 is localized to apical lumen in wild type and *pigv-1*(*qm34*) embryos. Note that the intestine of *pigv-1*(*qm34*) embryos is twisted at the initial segment close to the pharynx (insets). The images in the inset are deliberately shown in grey for clarity. A sum projection of embryo intestine is shown in panel A and C.
(TIF)

S7 Fig Abnormal excretory canal branching upon loss of GPI-APs. In wild type embryo, two pairs of tubules branch out from the cell body forming an H-shape excretory canal (top panel), whereas in *pigv-1*(*qm34*) embryo, more than two pairs of tubules branch out form cell body (middle and bottom panels) or branch out from the tubule itself (middle panel, indicated by a red arrowhead). CB stands for the excretory canal cell body. Numbers are written next to the tubules to highlight the difference in tubular branching between wild type and *pigv-1*(*qm34*) embryos. A maximum intensity projection of excretory canal marked by AQP-8::mCherry is shown in all panels.
(TIF)

S1 Movie Epidermal morphogenesis in wild type embryo expressing AJM-1::GFP. Movie starts from epidermal enclosure and ends halfway through elongation. Time is indicated in minutes.
(AVI)

S2 Movie Epidermal morphogenesis in *pigv-1*(*qm34*) embryo expressing AJM-1::GFP. Movie starts from epidermal enclosure and ends halfway through elongation. Note that ventral epidermal cells are misaligned, thus creates a gap between the cells from which the internal tissues leak out. Time is indicated in minutes.
(AVI)

S3 Movie Embryonic elongation in wild type embryo expressing HMP-1::GFP. Movie starts from the beginning of elongation and ends at the time of hatching. Time is indicated in minutes.
(AVI)

S4 Movie Embryonic elongation in *pigv-1*(*qm34*) embryo expressing HMP-1::GFP. Movie starts from the beginning of elongation. The embryo arrests during elongation due to the epithelial tissue rupture at the embryo anterior. Time is indicated in minutes.
(AVI)

S1 Table Temperature sensitivity of *pigv-1*(*qm34*) allele. (DOCX)

S2 Table Quantification of *pigv-1*(*qm34*) embryonic phenotypes. (DOCX)

S3 Table Embryonic lethality upon temperature upshift to 25^°^C or downshift to 15^°^C. (DOCX)

S4 Table Tissue specific rescue of *pigv-1*(*qm34*) allele. (DOCX)

S5 Table ERM-1::GFP rescue of *pigv-1*(*qm34*) allele. (DOCX)
