# Supplementary figures and images for: Glycosyl Phosphatidylinositol Anchor Biosynthesis Is Essential for Maintaining Epithelial Integrity during Caenorhabditis elegans Embryogenesis
Source: PLoS Genet. 2015 Mar 25;11(3):e1005082. doi: 10.1371/journal.pgen.1005082 (PMC4373761; doi:10.1371/journal.pgen.1005082)

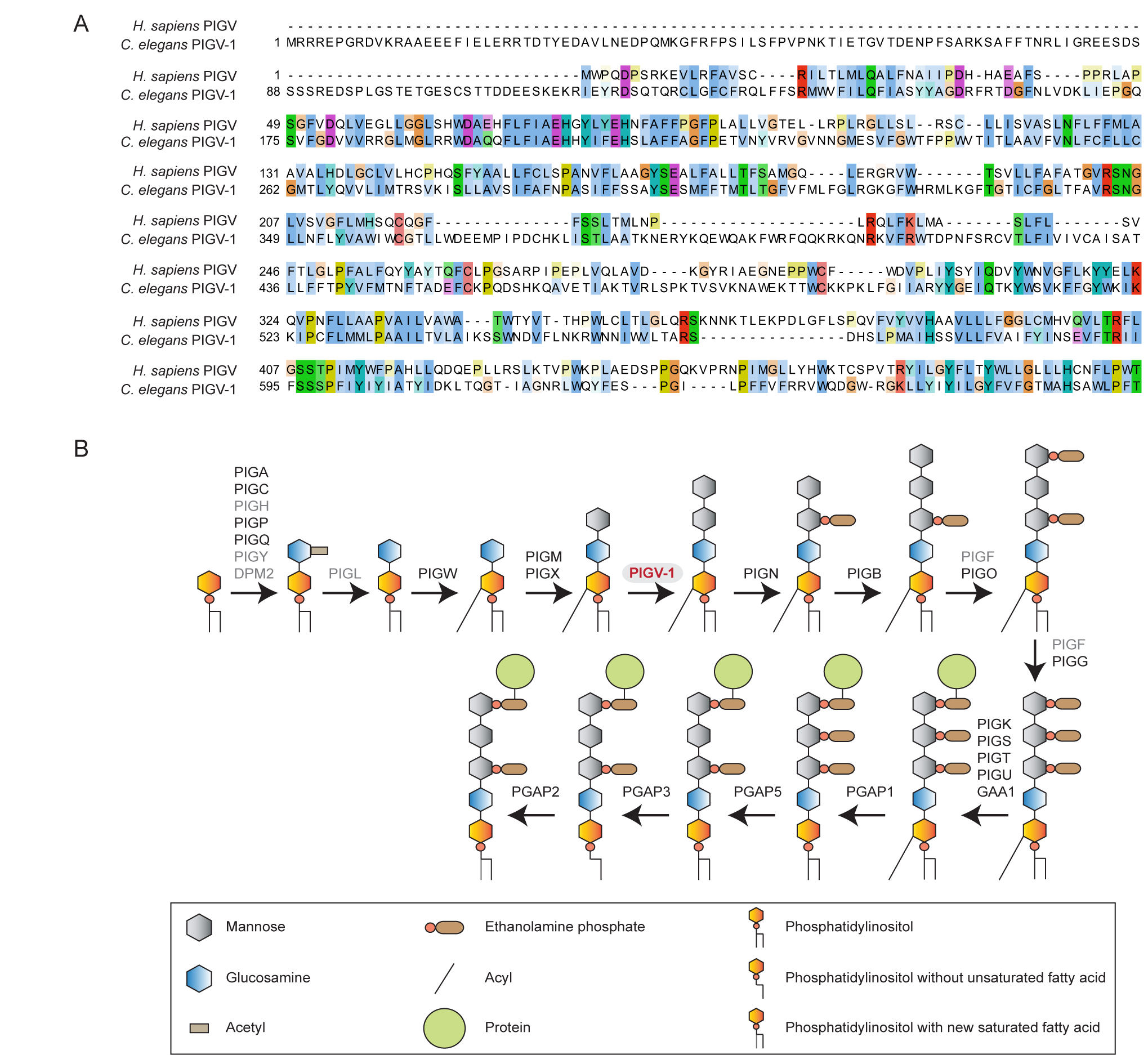

Supplement: S1 Fig — (A) An alignment of human PIGV and C. elegans PIGV-1 shows a high degree of similarity between them. Amino acid residues are colored based on ClustalX color scheme (http://www.jalview.org/help/html/colourSchemes/clustal.html) and displayed using Jalview. (B) The GPI-anchor biosynthesis pathway in mammalian cells. The orthologous enzymes present in C. elegans are shown in black and PIGV-1 whose mutant allele is assessed in this study is highlighted in red. (TIF) [file pgen.1005082.s001.tif]

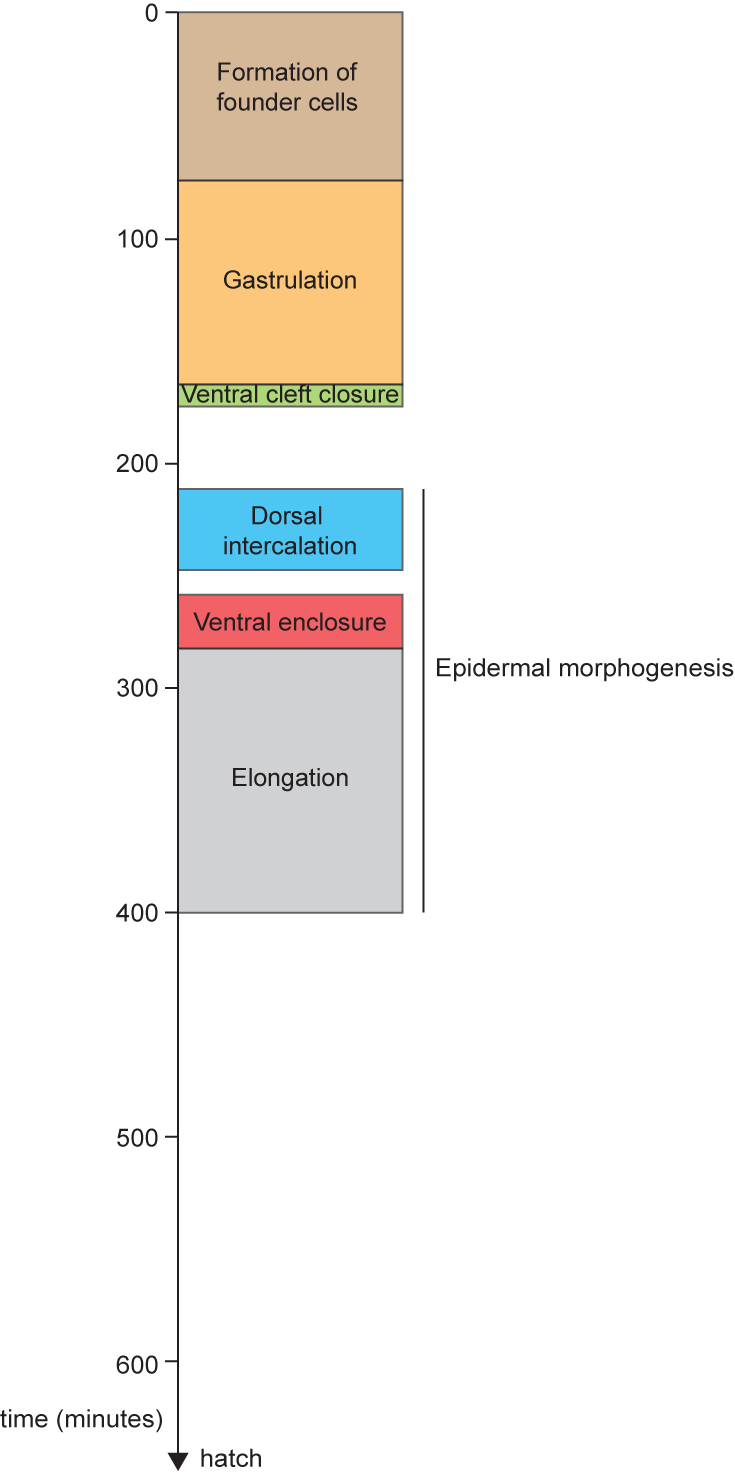

Supplement: S2 Fig — C. elegans embryogenesis is initiated by multiple rounds of cell divisions that form the founder cells. Three germ layers are then formed subsequently during gastrulation. The end of gastrulation is marked by closure of a ventral cleft. All those events occur within the first 2.5 hours of embryogenesis. In the next 4 hours, the embryo undergoes epidermal morphogenesis, in which epidermal cells born at the dorsal side of embryo intercalate and enclose the embryo at the ventral side. The embryo then elongates to form worm-like shape and hatches. (TIF) [file pgen.1005082.s002.tif]

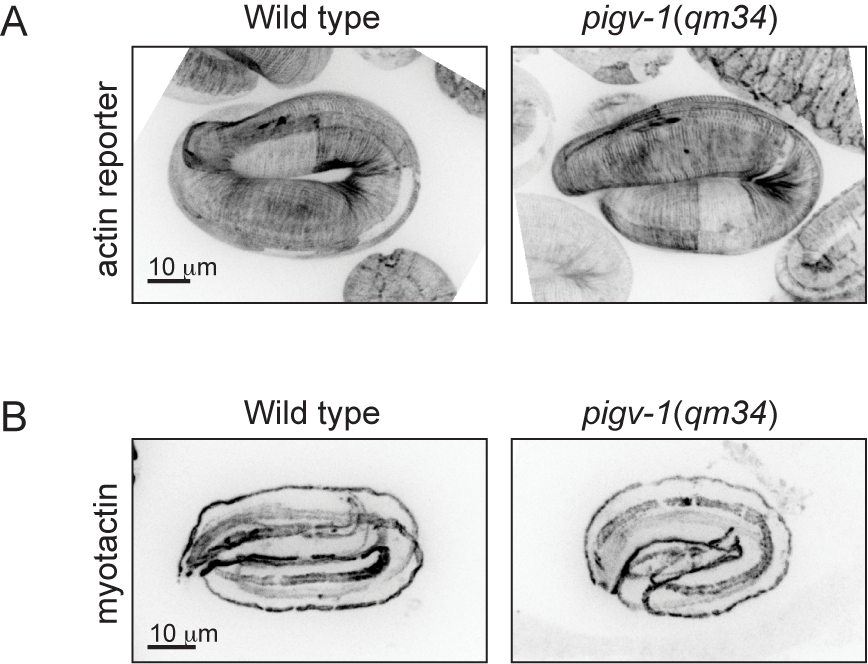

Supplement: S3 Fig — (A) Organization of circumferential actin bundles (CFBs) in pigv-1(qm34) embryo is similar to wild type embryo. VAB-10 actin binding domain tagged with GFP is used to visualize CFBs. (B) Muscle organization and structure are indistinguishable between wild type and pigv-1(qm34) embryos. Myotactin antibodies (MH46) are utilized to display muscles. A maximum intensity projection of CFBs and muscles are shown in panel A and B, respectively. (TIF) [file pgen.1005082.s003.tif]

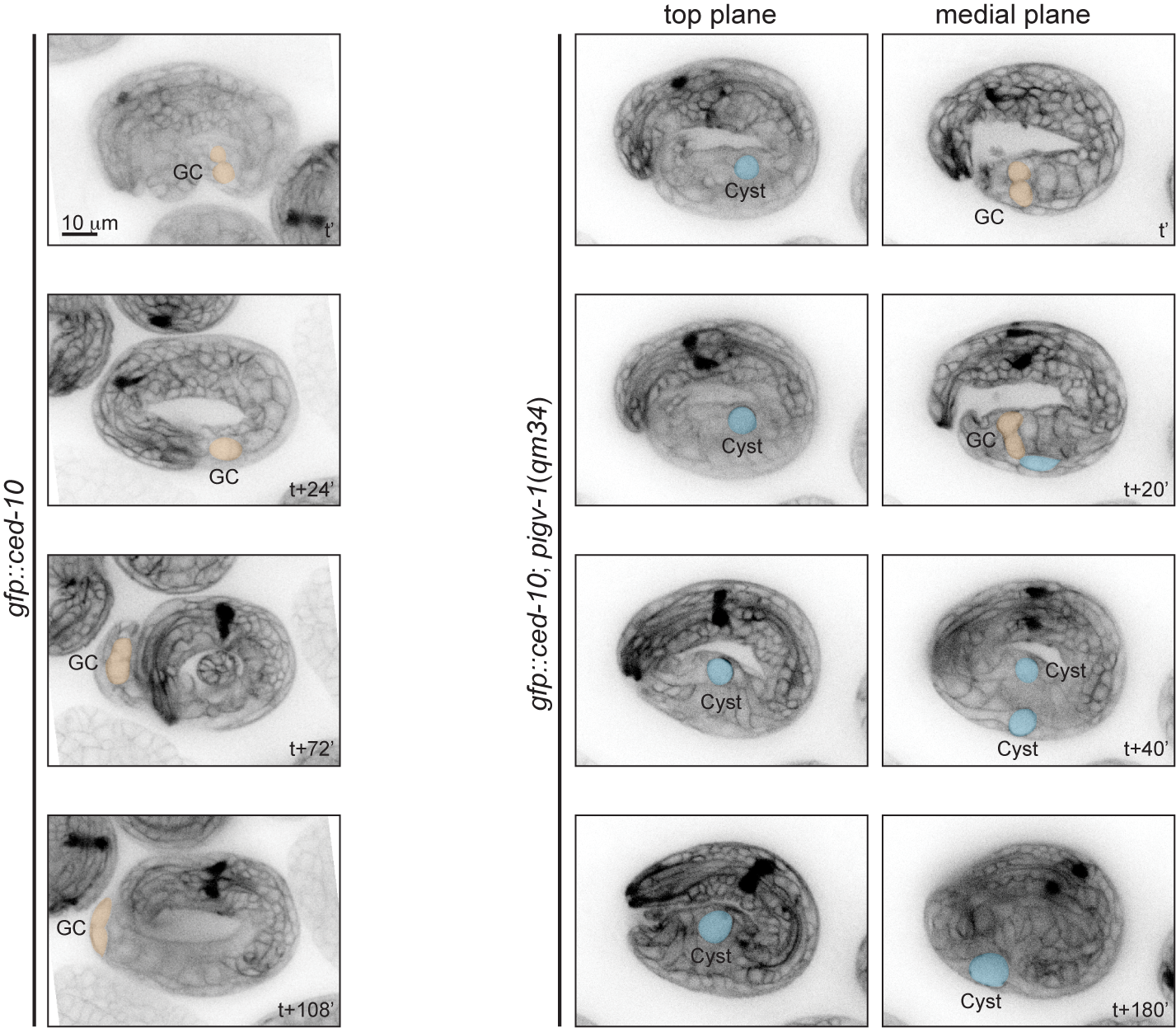

Supplement: S4 Fig — The germ cells (GC) are distinguishable from other cell types in the embryo based on their round shape and paired localization (left panels). The GCs are visible in pigv-1(qm34) embryo in a separate focal plane from that of the intestinal cysts (middle and right panels). The cysts in pigv-1(qm34) embryo grow in size over time. GCs are colored in orange and cysts are colored in blue. (TIF) [file pgen.1005082.s004.tif]

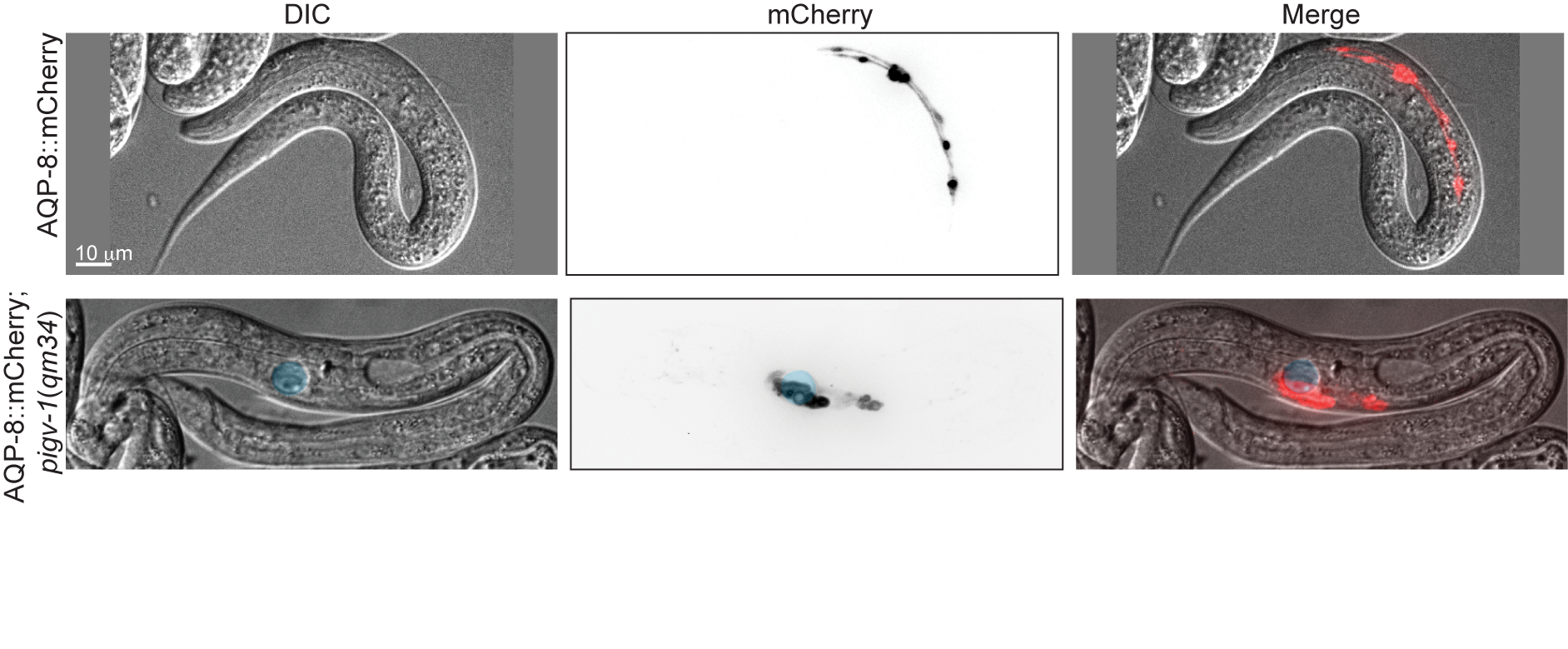

Supplement: S5 Fig — While the excretory canal in wild type L1 larva is elongated (top panel), the excretory canal in pigv-1(qm34) L1 larva is cystic and short (bottom panel). In both DIC and mCherry channels, cyst is colored in blue. (TIF) [file pgen.1005082.s005.tif]

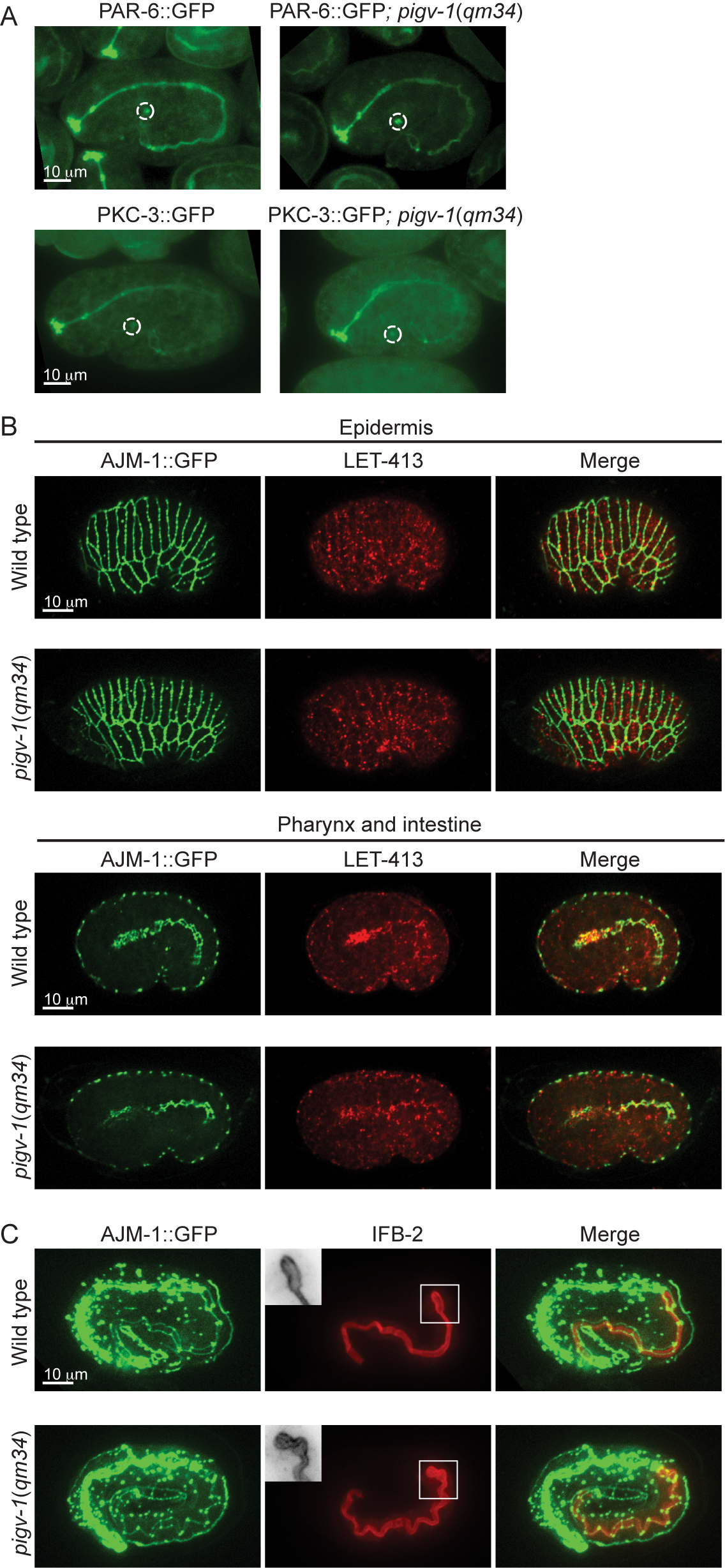

Supplement: S6 Fig — (A) GFP::PAR-6 and GFP::PKC-3 are localized to pharynx and intestine apical membranes both in wild type and pigv-1(qm34) embryos. Stippled circle denotes excretory cell. (B) AJM-1::GFP and LET-413 are correctly localized to apical and basolateral membrane of epidermis, pharynx and intestine, respectively, both in wild type and pigv-1(qm34) embryos. (C) IFB-2 is localized to apical lumen in wild type and pigv-1(qm34) embryos. Note that the intestine of pigv-1(qm34) embryos is twisted at the initial segment close to the pharynx (insets). The images in the inset are deliberately shown in grey for clarity. A sum projection of embryo intestine is shown in panel A and C. (TIF) [file pgen.1005082.s006.tif]

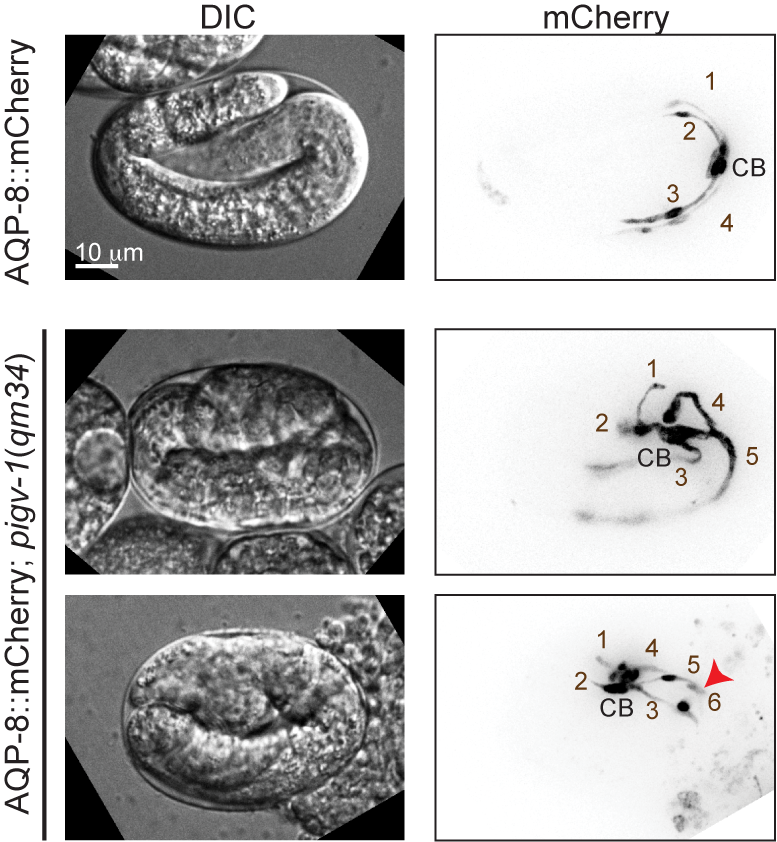

Supplement: S7 Fig — In wild type embryo, two pairs of tubules branch out from the cell body forming an H-shape excretory canal (top panel), whereas in pigv-1(qm34) embryo, more than two pairs of tubules branch out form cell body (middle and bottom panels) or branch out from the tubule itself (middle panel, indicated by a red arrowhead). CB stands for the excretory canal cell body. Numbers are written next to the tubules to highlight the difference in tubular branching between wild type and pigv-1(qm34) embryos. A maximum intensity projection of excretory canal marked by AQP-8::mCherry is shown in all panels. (TIF) [file pgen.1005082.s007.tif]
