# Supplementary material for: Glycosyl Phosphatidylinositol Anchor Biosynthesis Is Essential for Maintaining Epithelial Integrity during Caenorhabditis elegans Embryogenesis
Source: PLoS Genet. 2015 Mar 25;11(3):e1005082. doi: 10.1371/journal.pgen.1005082 (PMC4373761; doi:10.1371/journal.pgen.1005082)
Supplement: S1 Table — (DOCX) [file pgen.1005082.s012.docx]

**S1 Table. Temperature sensitivity of *pigv-1*(*qm34*) allele**

| Parental genotype  n ≥ 1000 embryos (≥ 100 animals) | % Embryonic lethality at 15^°^C | P values | % Embryonic lethality at 20^°^C | P values | % Embryonic lethality at 25^°^C | P values |
| --- | --- | --- | --- | --- | --- | --- |
| Wild type | 0.2 ± 0.5 | n.a. | 3.2 ± 1.8 | n.a. | 0.3 ± 0.6 | n.a. |
| *pigv-1*(*qm34*) | 1.8 ± 1.5 | 1 x 10^-1^ | 40 ± 15 | 1.3 x 10^-3^ | 82 ± 1.4 | 9 x 10^-14^ |

Average % embryonic lethality ± s.e.m. is indicated. Two-tailed Student’s *t*-test was applied to compare this value to the wild type.
