# Supplementary material for: Glycosyl Phosphatidylinositol Anchor Biosynthesis Is Essential for Maintaining Epithelial Integrity during Caenorhabditis elegans Embryogenesis
Source: PLoS Genet. 2015 Mar 25;11(3):e1005082. doi: 10.1371/journal.pgen.1005082 (PMC4373761; doi:10.1371/journal.pgen.1005082)
Supplement: S2 Table — (DOCX) [file pgen.1005082.s013.docx]

**S2 Table. Quantification of *pigv-1*(*qm34*) embryonic phenotypes**

| Parental genotype | n | % Embryonic lethality | | | | % Embryonic viability | | | |
| --- | --- | --- | --- | --- | --- | --- | --- | --- | --- |
|  |  | Without visible defect | With cysts and rupture | With cysts | With rupture | Without visible defect | With cysts and rupture | With cysts | With rupture |
| Wild type | 86 | 6 | 0 | 0 | 0 | 94 | 0 | 0 | 0 |
| *pigv-1*(*qm34*) | 176 | 8 | 26 | 1 | 56 | 1 | 2 | 0 | 6 |
| *pigv-1*(*qm34*); ERM-1::GFP | 159 | 15 | 8 | 6 | 25 | 36 | 2 | 2 | 6 |
