# Supplementary material for: Glycosyl Phosphatidylinositol Anchor Biosynthesis Is Essential for Maintaining Epithelial Integrity during Caenorhabditis elegans Embryogenesis
Source: PLoS Genet. 2015 Mar 25;11(3):e1005082. doi: 10.1371/journal.pgen.1005082 (PMC4373761; doi:10.1371/journal.pgen.1005082)
Supplement: S3 Table — (DOCX) [file pgen.1005082.s014.docx]

**S3 Table. Embryonic lethality upon temperature upshift to 25^°^C or downshift to 15^°^C**

| Embryogenesis stages  n ≥ 10 embryos | % Embryonic lethality at 25^°^C | % Embryonic lethality at 15^°^C |
| --- | --- | --- |
| Generation of founder cells | 100 | 14 |
| Gastrulation | 94 | 0 |
| Ventral cleft closure | 100 | 25 |
| Dorsal intercalation | 92 | 27 |
| Epidermal enclosure | 56 | 8 |
| Elongation early | 25 | 31 |
| Elongation late | 0 | 43 |
