# Supplementary material for: Glycosyl Phosphatidylinositol Anchor Biosynthesis Is Essential for Maintaining Epithelial Integrity during Caenorhabditis elegans Embryogenesis
Source: PLoS Genet. 2015 Mar 25;11(3):e1005082. doi: 10.1371/journal.pgen.1005082 (PMC4373761; doi:10.1371/journal.pgen.1005082)
Supplement: S4 Table — (DOCX) [file pgen.1005082.s015.docx]

**S4 Table. Tissue specific rescue of *pigv-1*(*qm34*) allele**

| Parental genotype  n ≥ 1000 embryos (≥ 100 animals) | % Embryonic lethality | P values |
| --- | --- | --- |
| Wild type | 0.3 ± 0.6 | n.a. |
| *pigv-1*(*qm34*) | 82 ± 1.4 | n.a. |
| *pigv-1*(*qm34*); fosmid WRM0638cC08 | 18 ± 4.3 | 2.9 x 10^-9^ |
| *pigv-1*(*qm34*); *pigv-1p::gfp::pigv-1* | 79 ± 3.2 | 1 x 10^-1^ |
| *pigv-1*(*qm34*); *lin-26p::gfp::pigv-1* | 78 ± 4.5 | 8.9 x 10^-2^ |
| *pigv-1*(*qm34*); *pha-4p::gfp::pigv-1* | 65 ± 4.5 | 1.1. x 10^-4^ |
| *pigv-1*(*qm34*); *aqp-8p::gfp::pigv-1* | 67 ± 8.7 | 9.1 x 10^-3^ |
| *pigv-1*(*qm34*); *erm-1p::gfp::pigv-1* | 20 ± 4.6 | 6.5 x 10^-9^ |

Average % embryonic lethality ± s.e.m. is indicated. Two-tailed Student’s *t*-test was applied to compare this value with that of *pigv-1*(*qm34*).
