# Supplementary material for: Glycosyl Phosphatidylinositol Anchor Biosynthesis Is Essential for Maintaining Epithelial Integrity during Caenorhabditis elegans Embryogenesis
Source: PLoS Genet. 2015 Mar 25;11(3):e1005082. doi: 10.1371/journal.pgen.1005082 (PMC4373761; doi:10.1371/journal.pgen.1005082)
Supplement: S5 Table — (DOCX) [file pgen.1005082.s016.docx]

**S5 Table. ERM-1::GFP rescue of *pigv-1*(*qm34*) allele**

| Parental genotype  n ≥ 1000 embryos (≥ 100 animals) | % Embryonic lethality | P values |
| --- | --- | --- |
| Wild type | 0.3 ± 0.6 | n.a. |
| *pigv-1*(*qm34*) | 82 ± 1.4 | n.a. |
| YFP::ACT-5 | 1.9 ± 1.8 | n.a. |
| YFP::ACT-5; *pigv-1*(*qm34*) | 85 ± 4.4 | 3.9 x 10^-1^ |
| ERM-1::GFP | 1.8 ± 1.2 | n.a. |
| ERM-1::GFP; *pigv-1*(*qm34*) | 56 ± 5 | 9.7 x 10^-6^ |
| ERM-1::GFP; *gfp*(*RNAi*) | 5.1 ± 2.5 | n.a. |
| ERM-1::GFP; *pigv-1*(*qm34*); *gfp*(*RNAi*) | 93 ± 2.4 | 1.2 x 10^-6^ |

Average % embryonic lethality ± s.e.m. is indicated. The first two-tailed Student’s *t*-test was applied to compare the values to that of *pigv-1*(*qm34*), whereas the last two-tailed Student’s *t*-test was applied to compare the value to that of ERM-1::GFP; *pigv-1*(*qm34*).
